# Optimal time intervals for vaginal breech births: a case-control study

**DOI:** 10.3310/nihropenres.13297.1

**Published:** 2022-08-04

**Authors:** Emma Spillane, Shawn Walker, Christine McCourt

**Affiliations:** 1 Maternity Services, Kingston NHS Foundation Trust, Kingston upon Thames, London, KT2 7QB, UK; 2 Women and Children's Health, King's College London, 10th floor North Wing, St Thomas' Hospital, London, SE1 7EH, UK; 3 Women and Children's Services, Chelsea and Westminster Hospital NHS Foundation Trust, 369 Fulham Road, London, SW10 9NH, UK; 4 Centre for Maternal & Child Health Research, City, University of London, 1 Myddleton Street, London, EC1R 1UB, UK

**Keywords:** Breech Presentation, Midwifery, Obstetrics, Medical Education, Case-Control, Delivery: Breech, Training

## Abstract

**Background:**

Breech births are associated with a high rate of hypoxic injury, in part due to cord occlusion during emergence. Maximum time intervals and guidelines oriented toward earlier intervention have been proposed in a Physiological Breech Birth Algorithm. We wished to further test and refine the Algorithm for use in a clinical trial.

**Methods:**

We conducted a retrospective case-control study conducted in a London teaching hospital, including 15 cases and 30 controls, during the period of April 2012 to April 2020. Our sample size was powered to test the hypothesis that exceeding recommended time limits is associated with neonatal admission or death. Data collected from intrapartum care records was analysed using SPSS v26 statistical software. Variables were intervals between the stages of labour and various stages of emergence (presenting part, buttocks, pelvis, arms, head). The chi-square test and odds ratios were used to determine association between exposure to the variables of interest and composite outcome. Multiple logistic regression was used to test the predictive value of delays defined as non-adherence the Algorithm.

**Results:**

Logistic regression modelling using the Algorithm time frames had an 86.8% accuracy, a sensitivity of 66.7% and a specificity of 92.3% for predicting the primary outcome. Delays between umbilicus and head >3 minutes (OR: 9.508 [95% CI: 1.390-65.046] *p*=0.022) and from buttocks on the perineum to head >7 minutes (OR: 6.682 [95% CI: 0.940-41.990] *p*=0.058) showed the most effect. Lengths of time until the first intervention were consistently longer among the cases. Delay in intervention was more common among cases than head or arm entrapment.

**Conclusion:**

Emergence taking longer than the limits recommended in the Physiological Breech Birth algorithm is predictive of adverse outcomes. Some of this delay is potentially avoidable. Improved recognition of the boundaries of normality in vaginal breech births may help improve outcomes.

## Plain English summary

When babies are born bottom-first, there is a risk that the baby could be starved of oxygen during the birth. To help prevent this, researchers developed a flowchart to guide when to help a baby out, the Physiological Breech Birth Algorithm. The first version was based on a study of actual breech birth videos and recommends that the birth should be complete within 7-5-3 minutes from buttocks-pelvis-umbilicus visible. This is different from current national guidance not to intervene until 5 minutes after the baby’s pelvis is born. We are using this new algorithm to guide midwives and doctors in the OptiBreech Care Trial, so we wanted to make sure it is safe and accurate.

## Introduction

Whilst vaginal breech birth accounts for only 0.3% of all births in the UK ^1^, it is overrepresented in cerebral palsy litigation costs, accounting for 12% of all claims ^2^. Awareness of this increased risk has led to a reliance on caesarean section (CS). Although occurring in only 3–4% of pregnancies, breech presentation is one of the leading causes for a first-time planned CS and associated risks in subsequent pregnancies ^3, 4^. However, a policy of universal 36-week ultrasound scans does not eliminate undiagnosed term breech presentation ^5^. As a majority of compensation claims occur following diagnosis late in labour ^2^, improving outcomes in these rarely occurring births to reduce the litigation burden remains an important concern. Additionally, some women wish to plan a vaginal breech birth and encounter reluctance from health care professionals who fear they cannot keep the birth safe ^6, 7^. 

Studies of vaginal breech birth outcomes frequently seek to identify risk factors associated with the mother or fetus that may increase the risk of a poor outcome, and guidelines often present a set of criteria which should be met before women are offered the option of a vaginal breech birth ^3^. However, even in studies with very large samples, clear associations between commonly accepted risk factors and adverse outcomes are not reliably demonstrated, with the consistent exception of birthweight <10 ^th^ centile ^8– 10^.

Although some of the increased risk is explained by underlying conditions which can cause the foetus to present in the breech position, the skill of the practitioner facilitating the vaginal breech birth is understood to have a significant effect on its safety ^3, 10, 11^. The components of what constitutes skilled practice, how these are developed and whether they might be modifiable to improve outcomes are less well understood. One of these components is thought to be an understanding of the mechanisms and physiology of a normal breech birth ^12^. Familiarity with these mechanisms underpins an ability to anticipate and avert complications, a marker of breech experience ^12^. Careful, evidence-based descriptions of what is ‘normal’ in breech births may therefore help more novice clinicians to anticipate and avert difficulty despite their lack of clinical experience. 

The available guidance on timings in late second stage (emergence) in vaginal breech births is inconsistent and largely based on professional opinion. The Royal College of Obstetricians and Gynaecologists 2017 guideline on the *Management of Breech Presentation* suggests that “intervention to expediate breech birth is required … if there is a delay of more than 5 minutes from delivery of the buttocks to the head, or of more than 3 minutes from the umbilicus to the head” ^3 (p17)^. The K2MS Perinatal Training Programme states, “The expected time interval between the birth of the baby’s bottom until the shoulders appear should be approximately 2 minutes.” ^13^ A 2009 textbook, Training in Obstetrics and Gynaecology: the essential curriculum, states: “The rule of ‘5’ has traditionally been used – 5 min for each of the three delivery stages: to umbilicus, rest of the body and shoulders, head.” ^14 (p278)^

Recent ability to analyse videos has created an opportunity to base such guidelines on evidence rather than assumption or tradition. Reitter *et al.*’s recent analysis of a cohort of upright (kneeling) VBB videos with good outcomes identified that in over 75% of births, the time interval between birth of the pelvis and head was under 3 minutes ^15^. However, it is not known whether intervals differ in cases of adverse outcomes, and if so, whether these delays are associated with unpreventable entrapment or avoidable delay in intervention. Further evidence is needed to develop robust guidance.

**Figure 1.**
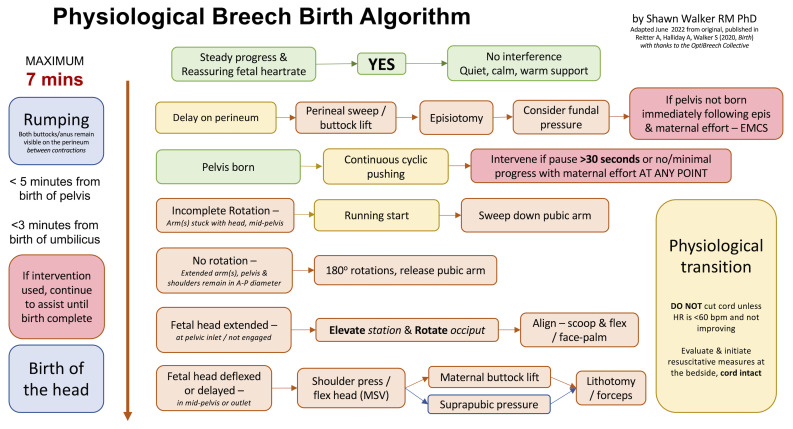
Physiological Breech Birth Algorithm. Designed by Shawn Walker RM PhD, June 2022 version. First published in Reitter A, Halliday A, Walker S, 2020, Practical insight into upright breech birth from birth videos: a structured analysis, Birth, 47(2):211–219.

Based on Reitter *et al.*’s 2020 analysis, a Physiological Breech Birth Algorithm was developed by Dr Shawn Walker and refined with feedback from professionals attending vaginal breech birth training. The most recent version is presented in Figure 1. Our study aimed to test the ability of the Algorithm to predict neonatal death or intensive care unit (NICU) admission among a retrospective sample of births in a London teaching hospital, based on whether the birth conformed to the guideline time frames in the Algorithm or not. We hoped to further verify or refine the Algorithm for use in a clinical trial.

## Methods

A single-centre retrospective case control study was conducted. The protocol defined cases as births where neonatal deaths or NICU admissions occurred (primary outcome). Controls were identified as the two vaginal breech births involving no neonatal death nor NICU admission, occurring directly prior to the identified case. Two previous births were used to prevent bias on the understanding that an adverse outcome can affect clinical decision-making for subsequent births ^16^. Any NICU admission was included because this indicates a neonate which requires additional observation, tests and/or intervention. Neonates who are not admitted are deemed as generally well ^17^. Neonatal admissions are very costly, and reducing avoidable admissions at term has been recognised as a priority ^18^. Additionally, separation from the baby was considered an important outcome by our Patient and Public Involvement (PPI) Group ^19^, who also requested more information on the timing of cord clamping.

To calculate our sample size, based on the work of Reitter *et al.*, ^15^ we hypothesised that the rate of exposure to a pelvis-to-head interval >3 minutes would be 25% among controls and 75% among cases. Using a case:control ratio of 1:2, we determined that 15 independent cases and 30 controls were required to infer an association between a pelvis-to-head interval >3 minutes and the composite neonatal outcome with a confidence interval of 95% and a power of 80%. We began seeking cases from the year 2020 and worked backward until the specified sample size was achieved.

The study was conducted within the maternity unit at a London District General Hospital which serves a large population of 176,313 people. Two thirds are of white British ethnicity and one third from Black, Asian and Minority Ethnic (BAME) backgrounds. The community the hospital serves is thought of as affluent, with good employment rates, particularly employment in high-end jobs. The hospital itself serves a wider community than the borough it is situated within and has 5000 births per year. It has a level two NICU situated within the maternity unit. The Algorithm was not in use at the site, and none of the authors were employed by the Trust, until 2020.

Our sample of 15 cases was achieved within the window of 2012 and 2020. These involved NICU admissions as no neonatal deaths occurred among the sample. A total of 71 term vaginal breech births were identified from routine electronic health records during this period. From this, we selected our 30 controls as the two vaginal breech births in the sample occurring immediately *prior* to each case. The Medical Record Numbers were sent to the Health Records Department for the complete files to be retrieved. Data were extracted by the lead researcher from the intrapartum care records and recorded anonymously in a Microsoft Excel spreadsheet.

A structured data collection tool was developed based on Reitter *et al.* ^13^ The data collection tool consisted of information usually recorded in the notes during a breech birth and included: lead professional, type of breech, position, epidural, fetal monitoring, meconium, what emerged first, time each part of the breech born, documented manoeuvres used, time performed and information related to the condition of the neonate at birth.

First, we calculated the time to event interval for variables of interest. We then reported descriptive statistics for all variables, including means, medians, absolute and interquartile ranges for continuous variables. Exposures and confounders were converted into binary variables, reflecting the guidance used in the Algorithm. These were then tested for association with the primary outcome using the non-parametric chi-square, or Fisher’s Exact tests where cell frequencies were too small for the chi-square test and odds ratios.

Logistic regression analysis was used to test the predictive values of meeting or exceeding the recommended time limits in the Physiological Breech Birth Algorithm. Logistic regression analyses were conducted with all variables that showed an association with the composite neonatal outcome to determine their predictive value, and additional variables to explore their potential as confounding factors for investigation in future studies. Finally, a Receiver Operating Characteristics (ROC) curve analysis was conducted to compare the sensitivity and specificity of the 7-5-3 minute time limit guidance. All statistical analyses were performed using IBM SPSS version 26.

This research was unfunded and was conducted as part of Spillane’s role as a Consultant Midwife with a remit for supporting vaginal breech births within the Trust. Spillane is also a Principal Investigator and Walker is the Chief Investigator for the OptiBreech Care Trial, an NIHR-funded feasibility study (NIHR300582, ISRCTN14521381) currently using the Physiological Breech Birth Algorithm. Two of the researchers have had a long-term involvement with the OptiBreech PPI group, who have experience of breech pregnancy and childbirth. Whilst the PPI group did not directly consider this study, their input into other aspects of the OptiBreech Project, including the prioritisation of outcomes, influenced our choices about variables of interest ^19^.

Approval was obtained through the Health Research Authority (IRAS 294936, 21/HRA/0562) and the Trust’s Research and Development department. This was a retrospective study using data that was anonymised by a member of the clinical care team; therefore, explicit consent was neither required nor sought.

## Results

**Figure 2.**
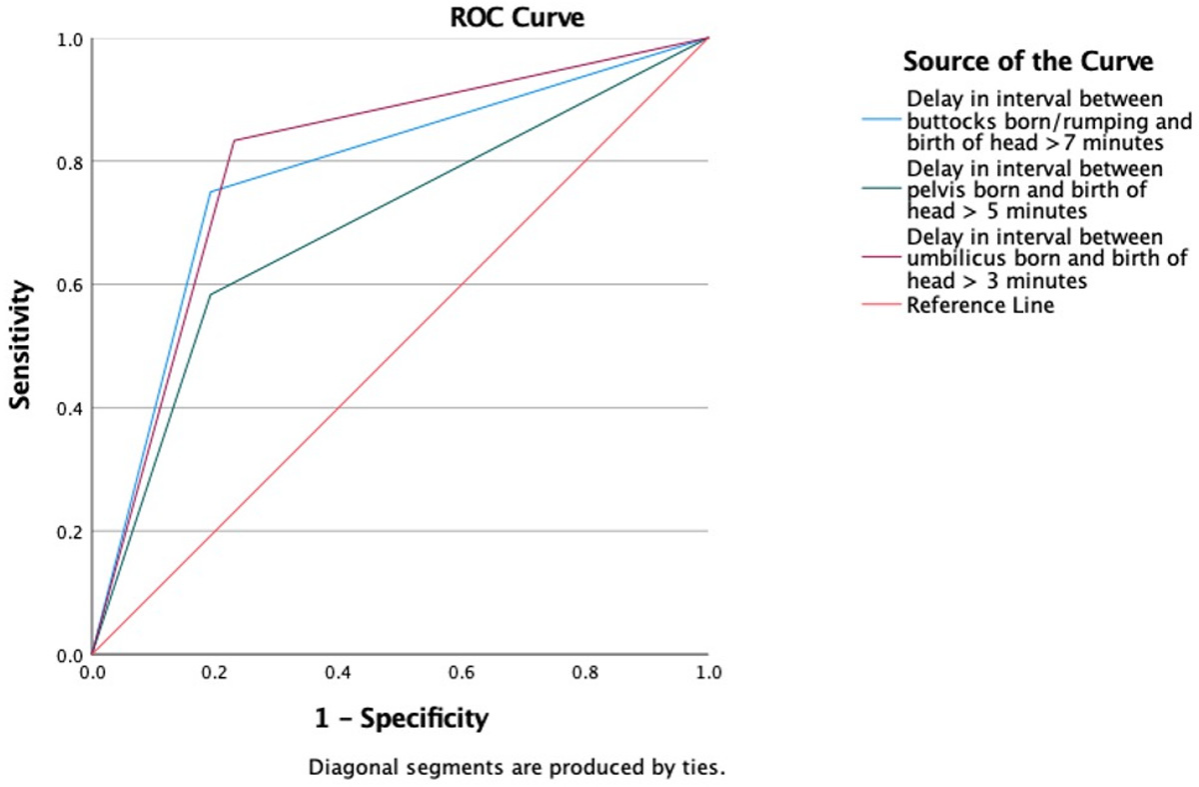
Predictive value of Physiological Breech Birth Algorithm’s 7-5-3 time limits.

The Physiological Breech Birth Algorithm reported in Reitter *et al.* ^15^ proposes three key interval limits: rumping(+3 station)-to-birth within 7 mins, pelvis-to-birth within 5 mins and umbilicus-to-birth within 3 mins. Our single-factor correlation tests showed that, in each of these categories, exceeding these limits was associated with NICU admission ( Table 1). When tested together in a logistic regression, the percentage accuracy (PAC) was 86.8%. The combination had a positive predictive value of 80.0% and sensitivity of 66.7%, and a negative predictive value of 85.7% and specificity of 92.3%. The most contributory factors predicting the primary outcome were an umbilicus-to-birth interval >3 minutes (aOR:9.508 [95% CI:1.390-65.046], *p*=0.022) and, to a lesser extent, a rumping-to-birth interval >7 minutes (aOR:6.282 [95% CI:0.940-41.990], *p*=0.058). The ROC curve is presented in Figure 2.

**Table 1.** Association of intrapartum risk factors with primary outcome (NICU admission or death). *P-values calculated with chi-square or Fisher’s exact test (2-sided)*.

Variable	Incidence of variable among controls vs cases	Odds Ratio	95% confidence interval	*p*-value
Length of second stage longer than 60 mins	6/30 vs 6/15	2.667	(.680 – 10.458)	.153
Length of second stage longer than 90 mins	3/30 vs 4/15	3.273	(.627 – 17.092)	.146
Rumping to birth exceeding 7 minutes	5/27 vs 10/14	11.000	(2.424 – 49.915)	.001
Pelvis born to birth exceeding 5 minutes	5/29 vs 8/13	7.680	(1.756 – 33.583)	.004
Pelvis born to birth exceeding 3 minutes	10/29 vs 13/13	*	*	<.0005
Umbilicus born to birth exceeding 3 minutes	8/29 vs 13/15	17.063	(3.127 – 93.106)	<.0005
Birth outside an obstetric unit	4/30 vs 2/15	1.00	(.161 – 6.192)	1.000
Birth facilitated by a midwife	18/30 vs 7/15	.583	(.167 – 2.036)	.396
Birth NOT facilitated by a senior registrar or consultant obstetrician	27/30 vs 7/15	.375	(.104 – 1.349)	.128
Non-extended breech presentation	6/30 vs 5/14	2.222	(.541 – 9.126)	.262
Intermittent monitoring	10/30 vs 1/14	.154	(.018 – 1.349)	.062
Meconium in labour	8/30 vs 6/15	1.833	(.494 – 6.810)	.362
Upright maternal birthing position	12/29 vs 7/15	1.240	(.353 – 4.348)	.737
Use of manual interventions	13/30 vs 15/15	*	*	<.0005
Use of epidural	6/30 vs 2/15	.615	(.108 – 3.495)	.581
Diagnosis after the start of labour	19/30 vs 12/15	2.316	(.534 – 10.041)	.255
Immediate cord clamping (<1 minute)	14/30 vs 14/14	*	*	.001

There was a statistically significant association between a pelvis-to-head interval of >3 minutes, the interval we used to calculate our sample size, and NICU admission following the birth ( *p*=<0.005). However, this result was highly confounded with an umbilicus-to-birth interval >3 minutes.

As expected, there was an association between use of manual interventions to assist the birth and the composite outcome of interest ( *p*=<.005). Manoeuvres were used in 13/30 controls and 15/15 cases. The intervals between the birth of the pelvis and the first manoeuvre used to assist the arms or head were twice as long in cases (mean 5.83, median 4, range 1–19 minutes) compared to controls (mean 2.45, median 2, range 0–6 minutes) ( Table 2). Where an episiotomy was performed, the interval between rumping and episiotomy was also longer in cases (mean 5.67, median 5, range 0–18 minutes) compared to controls (mean 1.75, median 1, range 0–5 minutes).

In both cases and controls, the mean and median reported times spent on manoeuvres to release the head were <1 minute. In only one case did the reported time exceed 2 minutes. In this case, 7 minutes were spent trying to release the head. However, intervals between the birth of the arms and initiation of manoeuvres to release the head were longer in cases (mean 2.42, median 2, range 1–5 minutes) than in controls (mean 1.20, median 1, range 0–2 minutes). In the case where head manoeuvres required 7 minutes, interventions were not attempted until 2 minutes after the arms were born and 13 minutes after the pelvis was born.

Similarly, in both cases and controls, the mean and median reported times spent on manoeuvres to release the arms were <1 minute. In one case, this took 3 minutes, and in one case it required 5 minutes. The intervals between the birth of the umbilicus and initiation of manoeuvres to release the arms were also longer in cases (mean 2.5, median 2, range 0–6 minutes) than in controls (mean .88, median 1, range 0–2 minutes).

In this sample, lengths of the first stage of labour were shorter for cases compared to controls. In contrast, the intervals between diagnosis of second stage of labour and birth were longer for cases compared to controls, as were the intervals between the onset of expulsive pushing and birth. Neither of these were significantly associated with NICU admission, either in single-factor analysis or logistic regression. Although 5-minute Apgar scores were lower for cases (mean 6.14, median 6) compared to controls (mean 9.76, median 10), arterial pH and base excess results did not differ.

An unexpected finding was that 28/44 (64%) of neonates experienced immediate umbilical cord clamping (UCC) following their breech births ( Table 1). This included 100% of the babies admitted to the neonatal unit, although in general the arterial cord blood gas results were marginally better among cases than controls ( Table 2). Mean arterial pH was 7.14 among controls vs 7.21 among cases, and mean base excess (BE) was -7.11 among controls compared to -6.65 among cases.

**Table 2.** Intervals of emergence and other variables.

Variable	Cases/Controls	Mean *mins*	Median *mins*	Minimum *mins*	Maximum *mins*	Inter-Quartile Range
Start of labour to diagnosis of second stage	Controls without interventions (17)	323	268	65	807	150 – 466
	Controls with interventions (13)	232	180	5	755	68 – 326
	Cases (14)	180	170	23	472	69 – 256
Diagnosis of second stage to birth	Controls without interventions (17)	46	27	6	163	15 – 64
	Controls with interventions (13)	42	28	9	137	18 – 52
	Cases (15)	74	49	7	294	18 – 97
Diagnosis of second stage to onset of active expulsive effort	Controls without interventions (17)	16	4	<1	91	<1 – 17
	Controls with interventions (13)	19	16	<1	78	<1 – 28
	Cases (15)	12	<1	<1	68	<1 – 25
Onset of active expulsive effort to birth	Controls without interventions (17)	31	22	6	77	11 – 53
	Controls with interventions (13)	23	13	4	117	9 – 22
	Cases (15)	61	31	7	294	13 – 91
Presenting part first visible to birth	Controls without interventions (17)	22	11	1	77	7 – 39
	Controls with interventions (13)	17	10	2	92	7 – 13
	Cases (14)	34	19	7	112	10 – 59
Rumping (buttocks born to +3 station) to birth	Controls without interventions (15)	4.5	3	1	13	1 – 7
	Controls with interventions (12)	7.5	6	2	32	3.3 – 7
	Cases (14)	15.5	10.5	5	55	7 – 19
Pelvis born to birth	Controls without interventions (16)	2.9	1.5	<1	10	1 – 5
	Controls with interventions (13)	3.6	3	1	7	2.5 – 5
	Cases (13)	8.8	7	4	22	5 – 9.5
Umbilicus born to birth	Controls without interventions (16)	2.2	1	<1	5	1 – 4
	Controls with interventions (13)	2.6	2	1	6	2 – 3.5
	Cases (15)	6.3	6	3	15	4 – 8
Arms born to birth	Controls without interventions (16)	1.3	1	<1	5	1 – 1
	Controls with interventions (11)	1.5	1	1	2	1 – 2
	Cases (14)	3.3	2.5	1	9	2 – 4
Rumping and episiotomy	Controls (4)	1.8	1	<1	5	<1 – 4.25
	Cases (9)	5.7	5	<1	18	<1 – 9
Birth of the pelvis to first manoeuvre to assist arms or head	Controls (11)	2.5	2	<1	6	1 – 4
	Cases (12)	5.8	4	1	19	2.25 – 7
Birth of the umbilicus to first manoeuvre to release the arms	Controls (8)	.9	1	<1	2	<1 – 1.75
	Cases (12)	2.5	2	<1	6	1 – 4.75
Birth of the arms to first manoeuvre to release the head	Controls (5)	1.2	1	<1	2	.5 – 2
	Cases (12)	2.4	2	1	5	2 – 3
Total time spent on manoeuvres to release the arms	Controls with interventions (8)	.38	<1	<1	2	<1 – 0.75
	Cases (12)	.83	<1	<1	5	0 – 1
Total time spent on manoeuvres to release the head	Controls with interventions (7)	.14	<1	<1	1	<1 – <1
	Cases (13)	.85	<1	<1	7	0 – 1
Arterial pH	Controls without interventions (2)	*	*	7.11	7.13	*
	Controls with interventions (8)	7.14	7.14	7.04	7.26	7.05 – 7.21
	Cases (6)	7.21	7.17	7.11	7.38	7.14 – 7.30
Arterial BE	Controls without interventions (2)	*	*	-9.3	-6.9	*
	Controls with interventions (8)	-7.11	-7.35	-10.9	-1.1	-10.38 – -4.53
	Cases (6)	-6.65	-7.40	-1.7	-11.6	-9.20 – -2.96
5-minute APGAR	Controls without interventions (16)	9.75	10	9	10	9 – 10
	Controls with interventions (13)	9.77	10	8	10	10 – 10
	Cases (14)	6.14	6	0	10	4.5 – 9
Timing of cord clamping	Controls without interventions (17)	9.29	1	<1	52	<1 – 12.5
	Controls with interventions (13)	2.46	<1	<1	28	<1 – 1
	Cases (14)	<1	<1	<1	<1	*

The data that support the findings of this study are openly available on Figshare, reference number 15134427.

## Discussion

### Main findings

Our findings demonstrate a relationship between NICU admission and longer time intervals around the time of emergence in vaginal breech births, including comparative delay in providing assistance. We found that the time limits described in the Physiological Breech Birth Algorithm, together, have a predictive accuracy (PAC) of 86.8%, with a sensitivity of 66.7% and specificity of 92.3%. Our findings therefore support the Algorithm’s guidance that it is ‘normal’ for vaginal breech births to be complete in under 7 minutes from rumping, under 5 minutes from the birth of the pelvis, and/or under 3 minutes from the birth of the umbilicus, including time for manual assistance.

Our findings support a more active approach to intervention in births that are not progressing swiftly once the breech begins to emerge. Delay in assisting appears likely to be a more significant contributing factor to neonatal compromise than head or arm entrapment. Regarding ability to predict no NICU admission, the true negative rate (92.3%) was higher than the ability to predict NICU admission, the true positive rate (66.7%). This suggests that adherence to Algorithm time frames is reassuring and safe but may result in unnecessary intervention in some births. Delay in response may be a modifiable contributing factor to poor outcomes in vaginal breech births, but it is not the only determinant.

The findings do not in any way suggest that manual interventions or episiotomy should be routine or immediate. Among controls, 17/30 births required no interventions. Application of manoeuvres prior to indication by delay or compromise could cause unintentional harm, just as much as hesitating to apply them. But the practice of instructing women to ‘breathe and wait for the next contraction’ should be abandoned ^20^. In the OptiBreech Trial, the guideline developed in consultation with our principal investigators indicates that, following any pause of 30 seconds or more, active maternal effort and movement (‘wiggle and push’) should be encouraged. This hands-off intervention can be used to confirm physical obstruction prior to use of manual interventions, to avoid iatrogenic harm.

### Strengths

This study uses rigorous scientific methods to demonstrate an association between delay in providing needed intervention and NICU admission following vaginal breech births. Reducing early separation from baby is an important outcome to service users and has significant economic implications. Building on video research used to develop the recommended (7-5-3) time limits ^15^, we formulated a plausible hypothesis that neonatal compromise would occur more often if the birth did not adhere to these. We then tested this hypothesis using a pre-specified sample size and found it was supported.

### Limitations

Our study’s sample size was determined to test a specific hypothesis, based on the pelvis-to-head interval identified in previous research ^15^; it was sufficient for this purpose but was insufficient to evaluate the influence of other intervals, such as the lengths of first and second stages of labour. For example, exploratory modelling indicated that first diagnosis during labour may have predictive value. This accords with case analysis of cerebral palsy litigation claims, in which breech presentation diagnosed late in labour was over-represented ^2^, but this factor was not associated with NICU admission in our single-factor analysis. Additionally, though we included neonatal death as part of a composite outcome, no neonatal deaths occurred during this sample period in this setting. Further research should use larger sample sizes to confirm or refute our results with smaller confidence intervals and test the influence of further variables.

While we collected very detailed information about factors not included in most studies, we did not collect information on other factors that are often noted to influence neonatal outcomes, for example, parity or fetal weight. Though neither the Term Breech Trial nor PREMODA studies indicated that parity or high fetal weight increased adverse outcomes ^9, 10^, these are often considered risk factors. They may be risk indicators instead, as both are more likely to increase exposure to delay in second stage, which if unaddressed may lead to harm. Prospective clinical studies are required to determine whether such delay is modifiable through changes in guidelines and training, and whether such changes improve outcomes or lead to other harms that delayed intervention helps to avoid.

This study used intrapartum care records, which are not always accurate. Reitter *et al.*’s study ^15^ analysed the timings around emergence in breech births using videos and could use data that were confirmed accurate to the second by two independent assessors. This study relied on documentation rounded to the nearest minute, which may have been recorded in retrospect if a scribe were not available at the time of birth. Systematic errors in the sample are likely to have been applicable to both cases and controls, and these results may change if data are collected prospectively.

### Interpretation

This research challenges some classical guidelines and beliefs concerning the intrapartum management of vaginal breech births. The RCOG guideline currently recommends that intervention is indicated at the point of a 5-minute delay following the birth of the pelvis. However, along with Reitter *et al.* ^15^, we have presented evidence that in most births with good outcomes, the head is born ***within*** 3 minutes of the birth of the pelvis; therefore intervention is indicated sooner. Our logistic regression analysis also indicated that the within 7-minutes-from-rumping interval may be a better overarching guideline interval.

It is also physiologically plausible that delays in the early stages of emergence contribute to the complication of head entrapment. Cord compression is likely once the breech reaches +3 station, when both buttocks and anus are visible on the perineum between contractions without recession. We refer to this as ‘rumping,’ the breech equivalent of crowning, after Evans ^21^. Delay at this point is likely to cause hypoxia and hypercapnia, leading to a loss of fetal tone and deflexion of the head and torso/arms, all of which ultimately make manual assistance more difficult. While ‘head entrapment’ is the complication so many clinicians dread, where it occurs 13 minutes after the birth of the pelvis, as in this study, head deflection due to hypoxia and poor tone is a more likely cause than cephalopelvic disproportion.

Many training programmes promote the maxim, “Hands off the Breech,” ^22, 23^ and suggest that touching of the baby could stimulate a startle response leading to arm or head entrapment ^3, 21^. While we agree that unskilled manipulation can cause harm, our findings suggest that delaying use of effective manoeuvres when indicated to assist the birth is also causing significant harm. Classical management strategies instruct trainees to ‘let the baby hang’ after the birth of the arms to assist head flexion and ‘wait until you see the nape of the baby’s neck.’ Our findings suggest that these instructions should be reconsidered or very carefully qualified. Clinicians need to understand how long they should wait before assisting the head into the pelvis if required, to avoid loss of situational awareness at this crucial point. Similarly, women should not be instructed to resist an urge to push and ‘wait for the next contraction’ without evidence that this improves outcomes.

A recently published evaluation gathered prospective outcome data following training based on the Algorithm ^24^. The evaluation included 90 vaginal breech births occurring in 6 NHS hospitals, with 21/90 births attended by someone who had completed the training. Among these, there were no severe adverse outcomes, compared to a rate of 7% among women (PPH >1500mL and OASIS) and 7% among neonates (5-minute Apgar <4 and NICU admission >4 days), where no one who had completed the training was present. The results of the evaluation can only be considered pilot data, but it remains the only evidence available of a training package that has demonstrated potentially improved outcomes for vaginal breech births using methods other than caesarean delivery.

The finding that cord blood gases were marginally worse among controls compared to cases may reflect the higher incidence of optimal cord clamping among this population. Although previous studies have reported changes (lower pH, higher BE) following delayed cord clamping, the differences we observed were larger than previously reported ^25, 26^, especially as nearly half of our controls also experienced immediate cord clamping. It seems likely that, in vaginal breech births, the high incidence of acute cord occlusion around the time of emergence (buttocks visible to birth) disrupts fetal gas exchange via the placenta. The blood captured within an immediately clamped umbilical cord may therefore reflect the fetal metabolic condition prior to the start of cord occlusion, rather than at birth. Cord blood taken from breech neonates at least 1 minute after birth may more accurately reflect the fetal metabolic condition at birth, as the fetal blood recirculates. Once the occlusion is relieved, the bradycardia caused by cessation of blood flow from the placenta to the heart recovers audibly in most neonates, with or without obvious respiratory effort.

For this reason, the finding that 64% of neonates in this sample experienced immediate UCC is also of concern. Current NICE ^27^ and Resuscitation Council ^28^ guidelines recommend clamping after at least 60 seconds wherever possible, and this should be standard management for all neonates where the fetal heart is >60bpm and rising with initial stimulation. At least 75% of these neonates had an Apgar score of 10 at 5 minutes and would likely have met this criterion at birth, if properly assessed. UCC is considered an important outcome by birthing women ^19^, who in general wish to prevent immediate UCC, and this priority is backed up by physiological evidence. UCC prior to the establishment of respiration in mildly hypoxic infants may initiate a reflex bradycardia and reduction in cardiac output, due to sudden cessation of blood flow returning to the heart. Such an ischemic insult may exacerbate any asphyxic insult ^29, 30^. For some time, due to service user input and a consensus of professionals experienced in physiological breech birth ^31^, our Algorithm has recommended initiation of resuscitation with the umbilical cord intact. We will continue to advocate for this approach, and have incorporated into the OptiBreech Care Trial guideline, as we continue to collect data on timing of UCC as both an outcome and an explanatory variable in outcomes for vaginal breech births.

Our case-control study suggests that skilled management around the time of emergence is a crucial factor in the safety of vaginal breech births. Differences in outcomes are apparent in a much smaller data set than those that have been used to define selection criteria, which appear to have a more negligible impact on outcomes. Despite the application of stricter selection criteria and consequent increase in the number of caesarean births for breech-presenting babies, rates of adverse outcomes for vaginal breech births themselves have not declined ^8^. Stricter selection criteria are unlikely to improve outcomes in the absence of critical changes to intrapartum guidelines, dissemination in training programmes and development of expertise within services ^32^. While some factors may be predictive of delay and/or need for intervention in late second stage, outcomes for births where delay occurs unpredictably will not improve without changes to the way professionals respond around the time of emergence.

## Conclusion

The Physiological Breech Birth Algorithm was originally designed to summarise and support training in the facilitation of breech births where the birthing person is in an upright position. However, the principles are applicable across all breech births, such as the sample in this study, which includes a mix of supine and upright birthing positions. The Algorithm’s principles include clear boundaries for ‘normality’ in vaginal breech births and indications for interventions based on variations of the mechanisms of breech birth. The manoeuvres required to resolve obstruction are also based on the same physiological principles, performed in slightly different ways depending on the birth position. We suggest that it could be more widely used as a framework for training and for evaluating adverse outcomes when they occur. Clinical outcomes associated with its use can be expected with reports of the OptiBreech Care Trial.

## Data availability

### Underlying data

Figshare: Optimal Time Intervals of Breech Births Dataset, https://doi.org/10.6084/m9.figshare.15134427.v1.

Data are available under the terms of the Creative Commons Zero "No rights reserved" data waiver (CC0 1.0 Public domain dedication).
