# Optimal time intervals for vaginal breech births: a case-control study [version 2; peer review: 1 approved, 2 approved with reservations, 1 not approved]

**DOI:** 10.3310/nihropenres.13297.1

**Published:** 2022-09-16

**Authors:** Emma Spillane, Shawn Walker, Christine McCourt

**Affiliations:** 1 Maternity Services, Kingston NHS Foundation Trust, Kingston upon Thames, London, KT2 7QB, UK; 2 Women and Children’s Health, King’s College London, 10th floor North Wing, St Thomas’ Hospital, London, SE1 7EH, UK; 3 Women and Children’s Services, Chelsea and Westminster Hospital NHS Foundation Trust, 369 Fulham Road, London, SW10 9NH, UK; 4 Centre for Maternal & Child Health Research, City, University of London, 1 Myddleton Street, London, EC1R 1UB, UK

**Keywords:** Breech Presentation, Midwifery, Obstetrics, Medical Education, Case-Control, Delivery: Breech, Training

## Abstract

**Background:**

Breech births are associated with a high rate of hypoxic injury, in part due to cord occlusion during emergence. Maximum time intervals and guidelines oriented toward earlier intervention have been proposed in a Physiological Breech Birth Algorithm. We wished to further test and refine the Algorithm for use in a clinical trial.

**Methods:**

We conducted a retrospective case-control study in a London teaching hospital, including 15 cases and 30 controls, during the period of April 2012 to April 2020. Our sample size was powered to test the hypothesis that exceeding recommended time limits is associated with neonatal admission or death. Data collected from intrapartum care records was analysed using SPSS v26 statistical software. Variables were intervals between the stages of labour and various stages of emergence (presenting part, buttocks, pelvis, arms, head). The chi-square test and odds ratios were used to determine association between exposure to the variables of interest and composite outcome. Multiple logistic regression was used to test the predictive value of delays defined as non-adherence the Algorithm.

**Results:**

Logistic regression modelling using the Algorithm time frames had an 86.8% accuracy, a sensitivity of 66.7% and a specificity of 92.3% for predicting the primary outcome. Delays between umbilicus and head >3 minutes (OR: 9.508 [95% CI: 1.390-65.046] *p*=0.022) and from buttocks on the perineum to head >7 minutes (OR: 6.682 [95% CI: 0.940-41.990] *p*=0.058) showed the most effect. Lengths of time until the first intervention were consistently longer among the cases. Delay in intervention was more common among cases than head or arm entrapment.

**Conclusion:**

Emergence taking longer than the limits recommended in the Physiological Breech Birth algorithm may be predictive of adverse outcomes. Some of this delay is potentially avoidable. Improved recognition of the boundaries of normality in vaginal breech births may help improve outcomes.

## Introduction

Whilst vaginal breech birth accounts for only 0.3% of all births in the UK^1^, it is overrepresented in cerebral palsy litigation costs, accounting for 12% of all claims^2^. Awareness of this increased risk has led to a reliance on caesarean section (CS). Although occurring in only 3–4% of pregnancies, breech presentation is one of the leading causes for a first-time planned CS and associated risks in subsequent pregnancies^3,4^. However, a policy of universal 36-week ultrasound scans does not eliminate undiagnosed term breech presentation^5^. As a majority of compensation claims occur following diagnosis late in labour^2^, improving outcomes in these rarely occurring births to reduce the litigation burden remains an important concern. Additionally, some women wish to plan a vaginal breech birth and encounter reluctance from health care professionals who fear they cannot keep the birth safe^6,7^.

Studies of vaginal breech birth outcomes frequently seek to identify risk factors associated with the mother or fetus that may increase the risk of a poor outcome, and guidelines often present a set of criteria which should be met before women are offered the option of a vaginal breech birth^3^. However, even in studies with very large samples, clear associations between commonly accepted risk factors and adverse outcomes are not reliably demonstrated, with the consistent exception of birthweight <10^th^ centile^8–10^.

Although some of the increased risk is explained by underlying conditions which can cause the foetus to present in the breech position, the skill of the practitioner facilitating the vaginal breech birth is understood to have a significant effect on its safety^3,10,11^. The components of what constitutes skilled practice, how these are developed and whether they might be modifiable to improve outcomes are less well understood. One of these components is thought to be an understanding of the mechanisms and physiology of a normal breech birth^12^. Familiarity with these mechanisms underpins an ability to anticipate and avert complications, a marker of breech experience^12^. Careful, evidence-based descriptions of what is ‘normal’ in breech births may therefore help more novice clinicians to anticipate and avert difficulty despite their lack of clinical experience.

The available guidance on timings in late second stage (emergence) in vaginal breech births is inconsistent and largely based on professional opinion. The Royal College of Obstetricians and Gynaecologists (RCOG) 2017 guideline on the *Management of Breech Presentation* suggests that “intervention to expedite breech birth is required … if there is a delay of more than 5 minutes from delivery of the buttocks to the head, or of more than 3 minutes from the umbilicus to the head”^3(p17)^. The K2MS Perinatal Training Programme states, “The expected time interval between the birth of the baby’s bottom until the shoulders appear should be approximately 2 minutes.”^13^ A 2009 textbook, Training in Obstetrics and Gynaecology: the essential curriculum, states: “The rule of ‘5’ has traditionally been used – 5 min for each of the three delivery stages: to umbilicus, rest of the body and shoulders, head.”^14(p278)^

Recent ability to analyse videos has created an opportunity to base such guidelines on evidence rather than assumption or tradition. Reitter *et al*.’s recent analysis of a cohort of upright (kneeling) vaginal breech birth videos with good outcomes identified that in over 75% of births, the time interval between birth of the pelvis and head was under 3 minutes^15^. However, it is not known whether intervals differ in cases of adverse outcomes, and if so, whether these delays are associated with unpreventable entrapment or avoidable delay in intervention. Further evidence is needed to develop robust guidance.

**Figure 1:**
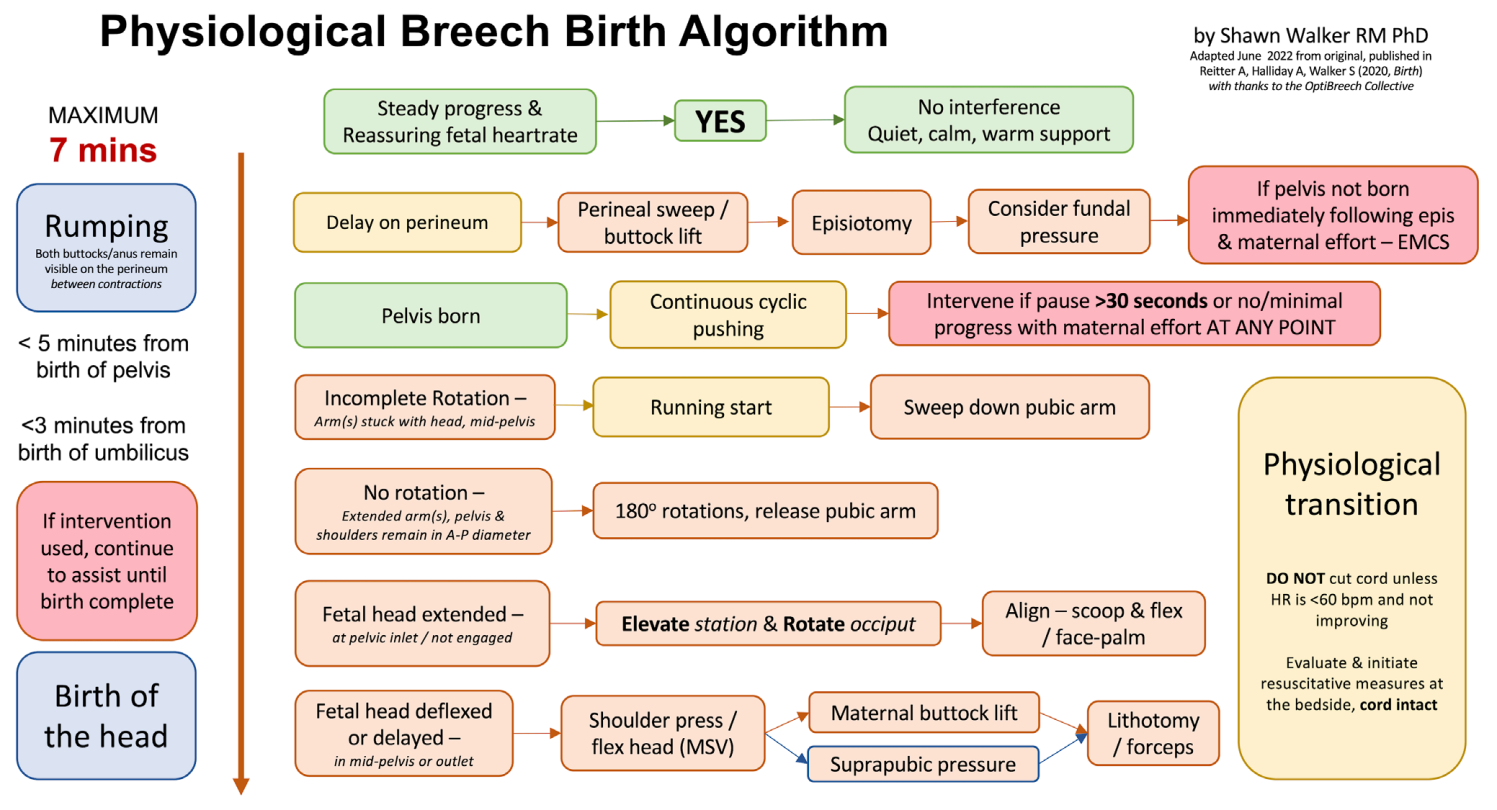
Physiological Breech Birth Algorithm. Designed by Shawn Walker RM PhD, June 2022 version. First published in Reitter A, Halliday A, Walker S, 2020, Practical insight into upright breech birth from birth videos: a structured analysis, Birth, 47(2):211–219.

Based on Reitter *et al*.’s 2020 analysis, a Physiological Breech Birth Algorithm was developed by Dr Shawn Walker and refined with feedback from professionals attending vaginal breech birth training. The most recent version is presented in Figure 1. Our study aimed to test the ability of the Algorithm to predict neonatal death or intensive care unit (NICU) admission among a retrospective sample of births in a London teaching hospital, based on whether the birth conformed to the guideline time frames in the Algorithm or not. We hoped to further verify or refine the Algorithm for use in a clinical trial.

## Methods

A single-centre retrospective case control study was conducted. The protocol defined cases as births where neonatal deaths or NICU admissions occurred (primary outcome). Controls were identified as the two vaginal breech births involving no neonatal death or NICU admission, occurring directly prior to the identified case. Two previous births were used to prevent bias on the understanding that an adverse outcome can affect clinical decision-making for subsequent births^16^. Any NICU admission was included because this indicates a neonate which requires additional observation, tests and/or intervention. Neonates who are not admitted are deemed as generally well^17^. Neonatal admissions are very costly, and reducing avoidable admissions at term has been recognised as a priority^18^. Additionally, separation from the baby was considered an important outcome by our Patient and Public Involvement (PPI) Group^19^, who also requested more information on the timing of cord clamping.

To calculate our sample size, based on the work of Reitter *et al*.,^15^ we hypothesised that the rate of exposure to a pelvis-to-head interval >3 minutes would be 25% among controls and 75% among cases. Using a case:control ratio of 1:2, we determined that 15 independent cases and 30 controls were required to infer an association between a pelvis-to-head interval >3 minutes and the composite neonatal outcome with a confidence interval of 95% and a power of 80%. We began seeking cases from the year 2020 and worked backward until the specified sample size was achieved.

The study was conducted within the maternity unit at a London District General Hospital which serves a large population of 176,313 people. Two thirds are of white British ethnicity and one third from Black, Asian and Minority Ethnic (BAME) backgrounds. The community the hospital serves is thought of as affluent, with good employment rates, particularly employment in high-end jobs. The hospital itself serves a wider community than the borough it is situated within and has 5000 births per year. It has a level two NICU situated within the maternity unit.

During the time period of the study (2012-2020), the hospital’s local guidance was based on current RCOG guidance^3,20^. All staff received annual mandatory training in obstetric emergencies, including a brief session on vaginal breech delivery. The Algorithm was first developed in 2017 and was not in general use at the site until 2021. A physiological breech birth training day was provided at the site in January 2018, which was attended by 39 members of staff. None of the authors were employed by the Trust, until 2020. The sample reflects a standard practice environment at the time, with mixed experience levels, and some staff having exposure to physiological breech birth theory and practice.

Our sample of 15 cases was achieved within the window of 2012 and 2020. These involved NICU admissions as no neonatal deaths occurred among the sample. A total of 71 term vaginal breech births were identified from routine electronic health records during this period. From this, we selected our 30 controls as the two vaginal breech births in the sample occurring immediately *prior* to each case. The Medical Record Numbers were sent to the Health Records Department for the complete files to be retrieved. Data were extracted by the lead researcher from the intrapartum care records and recorded anonymously in a Microsoft Excel spreadsheet.

A structured data collection tool was developed based on Reitter *et al*.^13^ The data collection tool consisted of information usually recorded in the notes during a breech birth and included: lead professional, type of breech, position, epidural, fetal monitoring, meconium, what emerged first, time each part of the breech born, documented manoeuvres used, time performed and information related to the condition of the neonate at birth. Data points were included if the information was clearly documented or could be reliably extrapolated. Some examples of this include: where the pelvis and head were born in the same minute, the umbilicus can reliably be assumed to have been born in the same minute as well; classification of rumping included any of the definitions used, both buttocks visible, anus visible, +3 station. Where data points could not be reliably discerned, they were not included in our analysis, and this is reflected in the denominators reported in the tables. Sometimes this information was extracted from risk reviews conducted following adverse outcomes, which recreated a timeline of events in detail based on notes and interviews with those in attendance.

First, we calculated the time to event interval for variables of interest. We then reported descriptive statistics for all variables, including means, medians, absolute and interquartile ranges for continuous variables. Exposures and confounders were converted into binary variables, reflecting the guidance used in the Algorithm. These were then tested for association with the primary outcome using the non-parametric chi-square, or Fisher’s Exact tests where cell frequencies were too small for the chi-square test and odds ratios.

Logistic regression analysis was used to test the predictive values of meeting or exceeding the recommended time limits in the Physiological Breech Birth Algorithm. Logistic regression analyses were conducted with all variables that showed an association with the composite neonatal outcome to determine their predictive value, and additional variables to explore their potential as confounding factors for investigation in future studies. Finally, a Receiver Operating Characteristics (ROC) curve analysis was conducted to compare the sensitivity and specificity of the 7-5-3 minute time limit guidance. All statistical analyses were performed using IBM SPSS version 26.

This research was unfunded and was conducted as part of Spillane’s role as a Consultant Midwife with a remit for supporting vaginal breech births within the Trust. Spillane is also a Principal Investigator and Walker is the Chief Investigator for the OptiBreech Care Trial, an NIHR-funded feasibility study (NIHR300582, ISRCTN14521381) currently using the Physiological Breech Birth Algorithm. Two of the researchers have had a long-term involvement with the OptiBreech PPI group, who have experience of breech pregnancy and childbirth. Whilst the PPI group did not directly consider this study, their input into other aspects of the OptiBreech Project, including the prioritisation of outcomes, influenced our choices about variables of interest^19^.

Approval was obtained through the Health Research Authority (IRAS 294936, 21/HRA/0562) and the Trust’s Research and Development department. This was a retrospective study using data that was anonymised by a member of the clinical care team; therefore, explicit consent was neither required nor sought.

## Results

**Figure 2:**
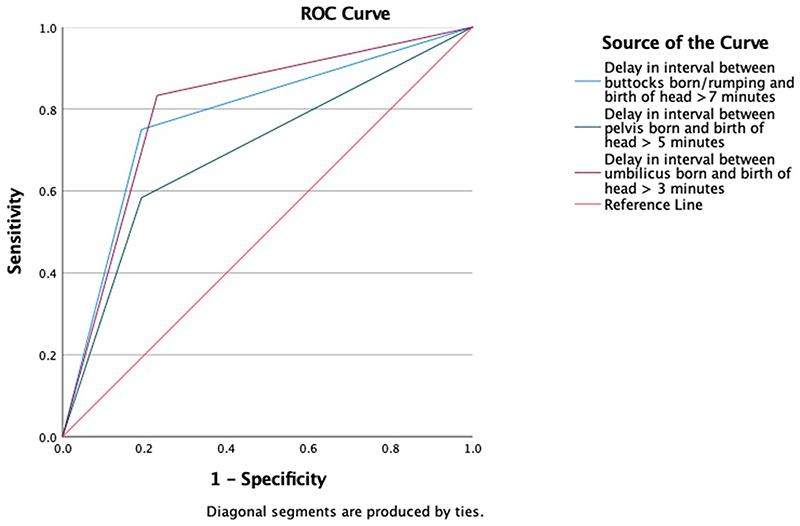
Predictive value of Physiological Breech Birth Algorithm’s 7-5-3 time limits.

The Physiological Breech Birth Algorithm reported in Reitter *et al*.^15^ proposes three key interval limits: rumping(+3 station)-to-birth within 7 mins, pelvis-to-birth within 5 mins and umbilicus-to-birth within 3 mins. Our single-factor correlation tests showed that, in each of these categories, exceeding these limits was associated with NICU admission (Table 1). When tested together in a logistic regression, the percentage accuracy (PAC) was 86.8%. The combination had a positive predictive value of 80.0% and sensitivity of 66.7%, and a negative predictive value of 85.7% and specificity of 92.3%. The most contributory factors predicting the primary outcome were an umbilicus-to-birth interval >3 minutes (aOR:9.508 [95% CI:1.390-65.046], *p*=0.022) and, to a lesser extent, a rumping-to-birth interval >7 minutes (aOR:6.282 [95% CI:0.940-41.990], *p*=0.058). The ROC curve is presented in Figure 2.

There was a statistically significant association between a pelvis-to-head interval of >3 minutes, the interval we used to calculate our sample size, and NICU admission following the birth (*p*=<0.005). However, this result was highly confounded with an umbilicus-to-birth interval >3 minutes.

As expected, there was an association between use of manual interventions to assist the birth and the composite outcome of interest (*p*=<.005). Manoeuvres were used in 13/30 controls and 15/15 cases. The intervals between the birth of the pelvis and the first manoeuvre used to assist the arms or head were twice as long in cases (mean 5.83, median 4, range 1–19 minutes) compared to controls (mean 2.45, median 2, range 0–6 minutes) (Table 2). Where an episiotomy was performed, the interval between rumping and episiotomy was also longer in cases (mean 5.67, median 5, range 0–18 minutes) compared to controls (mean 1.75, median 1, range 0–5 minutes).

In both cases and controls, the mean and median reported times spent on manoeuvres to release the head were <1 minute. In only one case did the reported time exceed 2 minutes. In this case, 7 minutes were spent trying to release the head. However, intervals between the birth of the arms and initiation of manoeuvres to release the head were longer in cases (mean 2.42, median 2, range 1–5 minutes) than in controls (mean 1.20, median 1, range 0–2 minutes). In the case where head manoeuvres required 7 minutes, interventions were not attempted until 2 minutes after the arms were born and 13 minutes after the pelvis was born.

Similarly, in both cases and controls, the mean and median reported times spent on manoeuvres to release the arms were <1 minute. In one case, this took 3 minutes, and in one case it required 5 minutes. The intervals between the birth of the umbilicus and initiation of manoeuvres to release the arms were also longer in cases (mean 2.5, median 2, range 0–6 minutes) than in controls (mean .88, median 1, range 0–2 minutes).

In this sample, lengths of the first stage of labour were shorter for cases compared to controls. In contrast, the intervals between diagnosis of second stage of labour and birth were longer for cases compared to controls, as were the intervals between the onset of expulsive pushing and birth. Neither of these were significantly associated with NICU admission, either in single-factor analysis or logistic regression. Although 5-minute Apgar scores were lower for cases (mean 6.14, median 6) compared to controls (mean 9.76, median 10), arterial pH and base excess results did not differ.

All admissions to the neonatal unit occurred following neonatal resuscitation, and this was documented as the reason for admission. An unexpected finding was that 28/44 (64%) of neonates experienced immediate umbilical cord clamping (UCC) following their breech births (Table 1). This included 100% of the babies admitted to the neonatal unit, although in general the arterial cord blood gas results were marginally better among cases than controls (Table 2). Mean arterial pH was 7.14 among controls vs 7.21 among cases, and mean base excess (BE) was -7.11 among controls compared to -6.65 among cases.

The data that support the findings of this study are openly available on Figshare, reference number 15134427.

## Discussion

### Main findings

Our findings demonstrate a relationship between NICU admission and longer time intervals around the time of emergence in vaginal breech births, including comparative delay in providing assistance. We found that the time limits described in the Physiological Breech Birth Algorithm, together, have a predictive accuracy (PAC) of 86.8%, with a sensitivity of 66.7% and specificity of 92.3%. Our findings therefore support the Algorithm’s guidance that it is ‘normal’ for vaginal breech births to be complete in under 7 minutes from rumping, under 5 minutes from the birth of the pelvis, and/or under 3 minutes from the birth of the umbilicus, including time for manual assistance.

Our findings support a active approach to intervention in births that are not progressing swiftly once the breech begins to emerge. Delay in assisting appears likely to be a more significant contributing factor to neonatal compromise than head or arm entrapment. Regarding ability to predict no NICU admission, the true negative rate (92.3%) was higher than the ability to predict NICU admission, the true positive rate (66.7%). This suggests that adherence to Algorithm time frames is reassuring and safe but may result in unnecessary intervention in some births. Delay in response may be a modifiable contributing factor to poor outcomes in vaginal breech births, but it is not the only determinant.

The findings do not in any way suggest that manual interventions or episiotomy should be routine or immediate. Among controls, 17/30 births required no interventions. Application of manoeuvres prior to indication by delay or compromise could cause unintentional harm, just as much as hesitating to apply them. But the practice of instructing women to ‘breathe and wait for the next contraction’ should be abandoned^21^. In the OptiBreech Trial, the guideline developed in consultation with our principal investigators indicates that, following any pause of 30 seconds or more, active maternal effort and movement (‘wiggle and push’) should be encouraged. This hands-off intervention can be used to confirm physical obstruction prior to use of manual interventions, to avoid iatrogenic harm.

### Strengths

This study uses rigorous scientific methods to demonstrate an association between delay in providing needed intervention and NICU admission following vaginal breech births. Reducing early separation from baby is an important outcome to service users and has significant economic implications. Building on video research used to develop the recommended (7-5-3) time limits^15^, we formulated a plausible hypothesis that neonatal compromise would occur more often if the birth did not adhere to these. We then tested this hypothesis using a pre-specified sample size and found it was supported.

### Limitations

Our study’s sample size was determined to test a specific hypothesis, based on the pelvis-to-head interval identified in previous research^15^; it was sufficient for this purpose but was insufficient to evaluate the influence of other intervals, such as the lengths of first and second stages of labour. For example, exploratory modelling indicated that first diagnosis during labour may have predictive value. This accords with case analysis of cerebral palsy litigation claims, in which breech presentation diagnosed late in labour was over-represented^2^, but this factor was not associated with NICU admission in our single-factor analysis. Additionally, though we included neonatal death as part of a composite outcome, no neonatal deaths occurred during this sample period in this setting. Further research should use larger sample sizes to confirm or refute our results with smaller confidence intervals and test the influence of further variables.

While we collected very detailed information about factors not included in most studies, we did not collect information on other factors that are often noted to influence neonatal outcomes, for example, parity or fetal weight. Though neither the Term Breech Trial nor PREMODA studies indicated that parity or high fetal weight increased adverse outcomes^9,10^, these are often considered risk factors. They may be risk indicators instead, as both are more likely to increase exposure to delay in second stage, which if unaddressed may lead to harm. Prospective clinical studies are required to determine whether such delay is modifiable through changes in guidelines and training, and whether such changes improve outcomes or lead to other harms that delayed intervention helps to avoid.

Neonatal admission was chosen as an outcome measure because it happens more often than severe neonatal outcomes, is costly and is an important consideration for service users, who prioritise avoiding early separation with the infant. Admission to a neonatal unit is often a subjective decision, as evidenced by the fact that in all cases of neonatal admission, the need for neonatal resuscitation was documented as the reason. Therefore, our data do not provide conclusive evidence of serious harm due to delay. However, lack of admission following births in which interventions were performed on average earlier provides some evidence that this does not necessarily result in an increase in harms due to trauma.

This study used intrapartum care records, which are not always accurate. Reitter *et al*.’s study^15^ analysed the timings around emergence in breech births using videos and could use data that were confirmed accurate to the second by two independent assessors. This study relied on documentation rounded to the nearest minute, which may have been recorded in retrospect if a scribe were not available at the time of birth. Systematic errors in the sample are likely to have been applicable to both cases and controls, and these results may change if data are collected prospectively.

### Interpretation

This research challenges some classical guidelines and beliefs concerning the intrapartum management of vaginal breech births. The RCOG guideline currently recommends that intervention is indicated at the point of a 5-minute delay following the birth of the pelvis. However, along with Reitter *et al*.^15^, we have presented evidence that in most births with good outcomes, the head is born ***within*** 3 minutes of the birth of the pelvis; therefore intervention is indicated sooner. Our logistic regression analysis also indicated that the within 7-minutes-from-rumping interval may be a better overarching guideline interval.

It is also physiologically plausible that delays in the early stages of emergence increase the likelihood of head entrapment. Cord compression is likely once the breech reaches +3 station, when both buttocks and anus are visible on the perineum between contractions without recession. We refer to this as ‘rumping,’ the breech equivalent of crowning, after Evans^21^. Delay at this point is likely to cause hypoxia and hypercapnia, leading to a loss of fetal tone and deflexion of the head and torso/arms, all of which ultimately make manual assistance more difficult. ‘Head entrapment’ is the complication so many clinicians dread. But where it occurs 13 minutes after the birth of the pelvis, as in this study, hypoxia and poor tone are likely contributors to head deflection. While delay at the end is often blamed for the poor outcome, it is often the last in a series of delays, some of which may be preventable.

Many training programmes promote the maxim, “Hands off the Breech,”^22,23^ and suggest that touching of the baby could stimulate a startle response leading to arm or head entrapment^3,24^. While we agree that unskilled manipulation can cause harm, our findings suggest that delaying use of effective manoeuvres when indicated to assist the birth is also causing significant harm. Classical management strategies instruct trainees to ‘let the baby hang’ after the birth of the arms to assist head flexion and ‘wait until you see the nape of the baby’s neck.’ Our findings suggest that these instructions should be reconsidered or very carefully qualified. Clinicians need to understand how long they should wait before assisting the head into the pelvis if required, to avoid loss of situational awareness at this crucial point. Similarly, women should not be instructed to resist an urge to push and ‘wait for the next contraction’ without evidence that this improves outcomes.

A recently published evaluation gathered prospective outcome data following training based on the Algorithm^25^. The evaluation included 90 vaginal breech births occurring in 6 NHS hospitals, with 21/90 births attended by someone who had completed the training. Among these, there were no severe adverse outcomes, compared to a rate of 7% among women (PPH >1500mL and OASIS) and 7% among neonates (5-minute Apgar <4 and NICU admission >4 days), where no one who had completed the training was present. The results of the evaluation can only be considered pilot data, but it remains the only evidence available of a training package that has demonstrated potentially improved outcomes for vaginal breech births using methods other than caesarean delivery.

The finding that cord blood gases were marginally worse among controls compared to cases may reflect the higher incidence of optimal cord clamping among this population. Although previous studies have reported changes (lower pH, higher BE) following delayed cord clamping, the differences we observed were larger than previously reported^26,27^, especially as nearly half of our controls also experienced immediate cord clamping. It seems likely that, in vaginal breech births, the high incidence of acute cord occlusion around the time of emergence (buttocks visible to birth) disrupts fetal gas exchange via the placenta. The blood captured within an immediately clamped umbilical cord may therefore reflect the fetal metabolic condition prior to the start of cord occlusion, rather than at birth. Cord blood taken from breech neonates at least 1 minute after birth may more accurately reflect the fetal metabolic condition at birth, as the fetal blood recirculates. Once the occlusion is relieved, the bradycardia caused by cessation of blood flow from the placenta to the heart recovers audibly in most neonates, with or without obvious respiratory effort.

For this reason, the finding that 64% of neonates in this sample experienced immediate UCC is also of concern. Current NICE^28^ and Resuscitation Council^29^ guidelines recommend clamping after at least 60 seconds wherever possible, and this should be standard management for all neonates where the fetal heart is >60bpm and rising with initial stimulation. At least 75% of these neonates had an Apgar score of 10 at 5 minutes and would likely have met this criterion at birth, if properly assessed. UCC is considered an important outcome by birthing women^19^, who in general wish to prevent immediate UCC, and this priority is backed up by physiological evidence. UCC prior to the establishment of respiration in mildly hypoxic infants may initiate a reflex bradycardia and reduction in cardiac output, due to sudden cessation of blood flow returning to the heart. Such an ischemic insult may exacerbate any asphyxic insult^30,31^. For some time, due to service user input and a consensus of professionals experienced in physiological breech birth^32^, our Algorithm has recommended initiation of resuscitation with the umbilical cord intact. We will continue to advocate for this approach, and have incorporated into the OptiBreech Care Trial guideline, as we continue to collect data on timing of UCC as both an outcome and an explanatory variable in outcomes for vaginal breech births.

Our case-control study suggests that skilled management around the time of emergence is a crucial factor in the safety of vaginal breech births. Differences in outcomes are apparent in a much smaller data set than those that have been used to define selection criteria, which appear to have a more negligible impact on outcomes. Despite the application of stricter selection criteria and consequent increase in the number of caesarean births for breech-presenting babies, rates of adverse outcomes for vaginal breech births themselves have not declined^8^. Stricter selection criteria are unlikely to improve outcomes in the absence of critical changes to intrapartum guidelines, dissemination in training programmes and development of expertise within services^33^. While some factors may be predictive of delay and/or need for intervention in late second stage, outcomes for births where delay occurs unpredictably will not improve without changes to the way professionals respond around the time of emergence.

## Conclusion

In this research, we have confirmed our hypothesis that an interval greater than 3 minutes between the birth of the fetal pelvis and the birth of the head is associated with neonatal admission or death. We have also demonstrated that births taking longer than the maximum parameters described in the Physiological Breech Birth Algorithm are predictive of neonatal admission or death. Questions remain about how often and at what point delay is associated with severe adverse outcomes, whether earlier intervention causes more harm than it prevents, and the role of umbilical cord clamp timing in mitigating some of the effects of hypoxia in vaginal breech births. We aim to explore these questions further in larger samples and a prospective study, which is currently on-going.

## Figures and Tables

**Table 1:** Association of intrapartum risk factors with primary outcome (NICU admission or death). *P-values calculated with chi-square or Fisher’s exact test (2-sided)*.

Variable	Incidence of variable among controls vs cases	Odds Ratio	95% confidence interval	p-value
Length of second stage longer than 60 mins	6/30 vs 6/15	2.667	(.680 – 10.458)	.153
Length of second stage longer than 90 mins	3/30 vs 4/15	3.273	(.627 – 17.092)	.146
Rumping to birth exceeding 7 minutes	5/27 vs 10/14	11.000	(2.424 – 49.915)	.001
Pelvis born to birth exceeding 5 minutes	5/29 vs 8/13	7.680	(1.756 – 33.583)	.004
Pelvis born to birth exceeding 3 minutes	10/29 vs 13/13	*	*	<.0005
Umbilicus born to birth exceeding 3 minutes	8/29 vs 13/15	17.063	(3.127 – 93.106)	<.0005
Birth outside an obstetric unit	4/30 vs 2/15	1.00	(.161 – 6.192)	1.000
Birth facilitated by a midwife	18/30 vs 7/15	.583	(.167 – 2.036)	.396
Birth NOT facilitated by a senior registrar or consultant obstetrician	27/30 vs 7/15	.375	(.104 – 1.349)	.128
Non-extended breech presentation	6/30 vs 5/14	2.222	(.541 – 9.126)	.262
Intermittent auscultation (vs continuous electronic fetal heart rate monitoring)	10/30 vs 1/14	.154	(.018 – 1.349)	.062
Meconium in labour	8/30 vs 6/15	1.833	(.494 – 6.810)	.362
Upright maternal birthing position	12/29 vs 7/15	1.240	(.353 – 4.348)	.737
Use of manual interventions	13/30 vs 15/15	*	*	<.0005
Use of epidural	6/30 vs 2/15	.615	(.108 – 3.495)	.581
Diagnosis after the start of labour	19/30 vs 12/15	2.316	(.534 – 10.041)	.255
Immediate cord clamping (<1 minute)	14/30 vs 14/14	*	*	.001

**Table 2:** Intervals of emergence and other variables.

Variable	Cases/Controls	Mean mins	Median mins	Minimum mins	Maximum mins	Inter-Quartile Range
Start of labour to diagnosis of second stage	Controls without interventions (17)	323	268	65	807	150 – 466
	Controls with interventions (13)	232	180	5	755	68 – 326
	Cases (14)	180	170	23	472	69 – 256
Diagnosis of second stage to birth	Controls without interventions (17)	46	27	6	163	15 – 64
	Controls with interventions (13)	42	28	9	137	18 – 52
	Cases (15)	74	49	7	294	18 – 97
Diagnosis of second stage to onset of active expulsive effort	Controls without interventions (17)	16	4	<1	91	<1 – 17
	Controls with interventions (13)	19	16	<1	78	<1 – 28
	Cases (15)	12	<1	<1	68	<1 – 25
Onset of active expulsive effort to birth	Controls without interventions (17)	31	22	6	77	11 – 53
	Controls with interventions (13)	23	13	4	117	9 – 22
	Cases (15)	61	31	7	294	13 – 91
Presenting part first visible to birth	Controls without interventions (17)	22	11	1	77	7 – 39
	Controls with interventions (13)	17	10	2	92	7 – 13
	Cases (14)	34	19	7	112	10 – 59
Rumping (buttocks born to +3 station) to birth	Controls without interventions (15)	4.5	3	1	13	1 – 7
	Controls with interventions (12)	7.5	6	2	32	3.3 – 7
	Cases (14)	15.5	10.5	5	55	7 – 19
Pelvis born to birth	Controls without interventions (16)	2.9	1.5	<1	10	1 – 5
	Controls with interventions (13)	3.6	3	1	7	2.5 – 5
	Cases (13)	8.8	7	4	22	5 – 9.5
Umbilicus born to birth	Controls without interventions (16)	2.2	1	<1	5	1 – 4
	Controls with interventions (13)	2.6	2	1	6	2 – 3.5
	Cases (15)	6.3	6	3	15	4 – 8
Arms born to birth	Controls without interventions (16)	1.3	1	<1	5	1 – 1
	Controls with interventions (11)	1.5	1	1	2	1 – 2
	Cases (14)	3.3	2.5	1	9	2 – 4
Rumping and episiotomy	Controls (4)	1.8	1	<1	5	<1 – 4.25
	Cases (9)	5.7	5	<1	18	<1 – 9
Birth of the pelvis to first manoeuvre to assist arms or head	Controls (11)	2.5	2	<1	6	1 – 4
	Cases (12)	5.8	4	1	19	2.25 – 7
Birth of the umbilicus to first manoeuvre to release the arms	Controls (8)	.9	1	<1	2	<1 – 1.75
	Cases (12)	2.5	2	<1	6	1 – 4.75
Birth of the arms to first manoeuvre to release the head	Controls (5)	1.2	1	<1	2	.5 – 2
	Cases (12)	2.4	2	1	5	2 – 3
Total time spent on manoeuvres to release the arms	Controls with interventions (8)	.38	<1	<1	2	<1 – 0.75
	Cases (12)	.83	<1	<1	5	0 – 1
Total time spent on manoeuvres to release the head	Controls with interventions (7)	.14	<1	<1	1	<1 – <1
	Cases (13)	.85	<1	<1	7	0 – 1
Arterial pH	Controls without interventions (2)	*	_*_	7.11	7.13	*
	Controls with interventions (8)	7.14	7.14	7.04	7.26	7.05 – 7.21
	Cases(6)	7.21	7.17	7.11	7.38	7.14 – 7.30
Arterial BE	Controls without interventions (2)	*	_*_	-9.3	-6.9	*
	Controls with interventions (8)	-7.11	-7.35	-10.9	-1.1	-10.38 – -4.53
	Cases(6)	-6.65	-7.40	-1.7	-11.6	-9.20 – -2.96
5-minute APGAR	Controls without interventions (16)	9.75	10	9	10	9 – 10
	Controls with interventions (13)	9.77	10	8	10	10 – 10
	Cases (14)	6.14	6	0	10	4.5 – 9
Timing of cord clamping	Controls without interventions (17)	9.29	1	<1	52	<1 – 12.5
	Controls with interventions (13)	2.46	<1	<1	28	<1 – 1
	Cases (14)	<1	<1	<1	<1	*

## Data Availability

Underlying data Figshare: Optimal Time Intervals of Breech Births Dataset, https://doi.org/10.6084/m9.figshare.15134427.v1. Data are available under the terms of the Creative Commons Zero “No rights reserved” data waiver (CC0 1.0 Public domain dedication).

## *REVISED*: Amendments from Version 1

We have updated the text of our original submission in response to peer review. Further information has been provided about breech training at the site during the study period. Clarification iss provided about how data was extracted from the notes, including any cases where this could be reliably extrapolated, and that missing information is reflected in the denominators in our tables. We have provided the further information that all neonatal admissions occurred following neonatal resuscitation, and that neonatal resuscitation was documented as the reason for admission in all cases. We have included further reflection on this as a subjective measure of neonatal well-being in our discussion, and how important it is to service users to minimise separation from the newborn around the time of birth. We have made minor revisions for clarity where reviewers indicated this would be useful. We have revised our conclusion to focus more clearly on providing a summary of the findings and the implications for our on-going research.

**Any further responses from the reviewers can be found at the end of the article**

## Plain english summary

When babies are born bottom-first, there is a risk that the baby could be starved of oxygen during the birth. To help prevent this, researchers developed a flowchart to guide when to help a baby out, the Physiological Breech Birth Algorithm. The first version was based on a study of actual breech birth videos and recommends that the birth should be complete within 7-5-3 minutes from buttocks-pelvis-umbilicus visible. This is different from current national guidance not to intervene until 5 minutes after the baby’s pelvis is born. We are using this new algorithm to guide midwives and doctors in the OptiBreech Care Trial, so we wanted to make sure it is safe and accurate.

## Open Peer Review

Current Peer Review Status: ✖ ✔ ？ ？

**Version 2**

**Reviewer Report 11 January 2023**

https://doi.org/10.3310/nihropenres.14441.r28863

**© 2023 Bahl R.** This is an open access peer review report distributed under the terms of the Creative Commons Attribution License, which permits unrestricted use, distribution, and reproduction in any medium, provided the original work is properly cited.

？ **Rachna Bahl**

Department of Obstetrics and Gynaecology, St. Michael's Hospital, University of Bristol, Bristol, UK

This is an important study that challenges the traditional teaching of breech birth. This is also the first study of this kind.

1. My main concern is the strength of recommendation in paragraph 3 of the discussion section. That its based on a review of 12 cases of breech births leading to NICU admissions. The data is retrospective and the notes are often written after the birth and maybe influenced by the outcome. The number of breech births is small overall. I would suggest rephrasing the paragraph.
2. I am aware that there is a highly skilled team of midwives performing these births. It would be interesting to know the experience of midwives conducting the births in this cohort. Often in obstetrics the skill of person making decisions and performing the action is very important to the outcome.
3. The authors have justified not taking parity and the birth weight into account. Whilst I agree with their reasons, I do believe that a difference in parity needs to be excluded.
4. Can the authors provide information on duration of NICU stay and the final diagnosis? Also were there babies from the control group who were subsequently admitted to NICU? This is important given worse pH in the control group.
5. The conclusion of the paper talks about a 3 min interval between birth of pelvis and head. The protocol says 5 min. Kindly adhere to the timings in the protocol and the results section of the abstract throughout the manuscript.
6. Table 2 gives units for cord bloods and Apgars as minutes. Please amend.

**Is the work clearly and accurately presented and does it cite the current literature?**

Yes

**Is the study design appropriate and is the work technically sound?**

Partly

**Are sufficient details of methods and analysis provided to allow replication by others?**

Yes

**If applicable, is the statistical analysis and its interpretation appropriate?**

I cannot comment. A qualified statistician is required.

**Are all the source data underlying the results available to ensure full reproducibility?**

Partly

**Are the conclusions drawn adequately supported by the results?**

Yes

***Reviewer Expertise:*** Intrapartum care

**I confirm that I have read this submission and believe that I have an appropriate level of expertise to confirm that it is of an acceptable scientific standard, however I have significant reservations, as outlined above.**

**Reviewer Report 20 December 2022**

https://doi.org/10.3310/nihropenres.14441.r28865

**© 2022 Bovbjerg M.** This is an open access peer review report distributed under the terms of the Creative Commons Attribution License, which permits unrestricted use, distribution, and reproduction in any medium, provided the original work is properly cited.

？ **Marit L. Bovbjerg**^iD^

Epidemiology Program, College of Public Health and Human Sciences, Oregon State University, Corvallis, OR, USA

This is a case control study designed to assess timing standards and protocols for vaginal breech birth, in terms of reducing NICU admissions. I applaud the authors for publishing this manuscript open access; vaginal breech is a common enough occurrence that we should know how to do it well. The sample size is very small, and as currently written some of the methods are either not well explained or not exactly correct. The manuscript is well-written.

Abstract

○ Please clarify who are 'cases' and 'controls'. I started with the abstract, and assumed cases would be neonates who experienced birth related hypoxia. But no, from the methods section, they're kids who were in the NICU or dead. The latter two of which are great patient-centered outcomes! Suggest (here and in the intro) therefore not starting the whole thing out with "hypoxic injury" then--it seems to the reader like that will be the main issue. But no. The main issue is seriously brain damaged/dead babies. Start there, and then say "secondary to hypoxia during birth" at some point.

Introduction

○ Well written. Clear. Consise.
○ However, as I say above under 'abstract' suggest not leading off with hypoxia, since that's not really the issue. That's a mediator, on the road to brain damage/death.
○ my only other suggestion is to strengthen the objective statement and actually say what you're doing. Instead of "we hoped to" say "our objective was to"
○ And please make it SPECIFIC! "seeing whether this thing we published previously is correct" is not specific. Seeing whether a given cutoff is the best one is more specific. Defining optimal times for intervention is specific. etc.

Methods

○ Thanks for including a power calculation! A bigger sample would have been better, of course, bc those confidence intervals in table 1 are awfully wide, and many of them cross 1. More clarity could come with a bigger sample; but I understand you are using this algorithm for an upcoming larger trial, so perhaps you can repeat this analysis with that larger sample.
○ Please clarify sampling procedure. If what you did is
  ○ take all the vaginal breech births
    ○ but starting from when in labor? All who were in second stage with a breech fetus? So planned vaginal breech who head for cesarean during first stage are out? Or something else? What are that hospital's protocols for breech? Who can plan one? Who heads straight for the OR with unplanned (eg, I assume footling/knee are out…also primips?)
  ○ of those vaginal breech births, find the first 15 you could who ended up in the NICU (or dead, but there weren't any of those)
    ○ were there the a priori time frames on those? eg, NICU within the first 24 hours? Dead in the first week?
  ○ then took the previous two vaginal breeches that did NOT result in a case
    ○ but what if they did? Like, what if there had been a breech/NICU, then the previous vaginal breech was ALSO a NICU? Then who would the controls have been? I'm guessing it didn't come up, but what was your plan if it had?
○ Please clarify what a "ROC curve analysis" is. You drew one, then….used Youden's index? calculated the area under the curve? (in which case, why do logistic regressions?)
○ Given extremely small sample size, logistic regressions aren't really OK with more than 2-3 dichotomous covariables. That will overfit the models, and make them less likely to apply to additional/external samples.
○ I don't understand what you did with logistic regressions. If it's "predictive values of meeting or exceeding the time limit"…so then the outcome of your logistic regression is NICU/death, and the exposure is ">= time limit", and you threw in a couple potential confounders? That's not really a "predictive value" (which since you're also doing ROC curves, PPV and NPV are things related to sensitivity and specificity)

Results

○ Is PAC just the area under the ROC curve?
○ Table 1 - why are there asterisks for three of the outcomes? I first assumed bc there were 0 cells in the 2x2 table, but no, there were plenty of events?
○ The ROC curve--ah. I'm not sure this is useful, given each "test" had only one possible cutpoint. Usually ROC curves are for comparing multiple possible cutpoints (eg, rumping to birth 5 min, 6 min, 7 min, 8 min, 9 min), and/or for area under the curve, which is overall discriminatory power, but again interpretation assumes multiple possible cutpoints.
○ Please discuss PPV and NPV separately from sens/spec, since the former will vary based on prevalence of NICU admission in a given sample
○ Given opening paragraph, I'm not sure what you did, methods-wise. Put all three timepoints (rumping, pelvis, umbilicus) into the model, as independent predictors, each as a yes/no variable given the 3/5/7 cutoffs? OK. So no confounders? (good, bc your sample is too small)
○ Second paragraph of results…wait, so you did test multiple possible cutpoints? Here it mentions >3 min for pelvis and >3 for umbilicus? Please clarify, the logistic regressions and the ROC curve analyses
○ Table 2 - did all cases have interventions? If not, please differentiate in the table. If yes, please make that as a separate point.
○ Towards the end, the parts about how long before interventions--that needs to be mentioned more in the methods. There's just the one sentence about calculating time to event intervals. Also, please say calculate time to interventions, and then we compared time to interventions by..
○ A time-line-y figure with times to interventions, say, with different colors for cases and controls, would really make your point about intervening earlier

Discussion

○ Given that I don't think you explored other time limits, you can't say it's "normal" for THIS to happen in under THAT amount of time. It might be better at 6 min 30 sec, say. (Also suggest avoiding "normal" as a word to mean anything other than it's statistical definition)
○ If your first sentence under strengths is about delays in intervening…then maybe that's the overall study objective?
○ Second paragraph in interpretation--Your data doesn't say anything about poor tone/deflection, nor 13 minutes for head birth…that I saw? If this is referencing something else, please cite. Otherwise, please don't speculate beyond your data.
○ I like your critique of current thinking (hands off, let it hang, etc)--we should be using data!

Minor points:

○ Second paragraph of the introduction, last sentence--do you mean birthweights greater than the 90^th^ percentile? I thought it was macrosomic babies who didn't do so hot with vaginal breech. Not the little ones.
○ "rumping" in lieu of "crowning" in your algorithm cracks me up
○ "BAME" is absolutely not how we would write about minoritized people in the USA, but if it's a standard UK term then fine
○ Consider not identifying the exact hospital, especially given you have a study sample explicitly selected bc of poor outcomes. people might be identifiable, if they've told their friends details, for instance. (wait, there's only NICUs, no deaths. Not as bad, then. But still…consider just saying "a large urban hospital in the UK with 5000 births per year")

**Is the work clearly and accurately presented and does it cite the current literature?**

Partly

**Is the study design appropriate and is the work technically sound?**

Partly

**Are sufficient details of methods and analysis provided to allow replication by others?**

No

**If applicable, is the statistical analysis and its interpretation appropriate?**

Partly

**Are all the source data underlying the results available to ensure full reproducibility?**

No

**Are the conclusions drawn adequately supported by the results?**

Yes

***Reviewer Expertise:*** perinatal epidemiology; maternity care health services research

**I confirm that I have read this submission and believe that I have an appropriate level of expertise to confirm that it is of an acceptable scientific standard, however I have significant reservations, as outlined above.**

**Reviewer Report 31 October 2022**

https://doi.org/10.3310/nihropenres.14441.r28784

**© 2022 Bogner G.** This is an open access peer review report distributed under the terms of the Creative Commons Attribution License, which permits unrestricted use, distribution, and reproduction in any medium, provided the original work is properly cited.

✖ **Gerhard Bogner**^iD^

Department of Obstetrics and Gynaecology, Paracelsus Medical University, Salzburg, Austria

Unfortunately, I am not yet convinced by the author's answer ("Clarification is provided about how data was extracted from the notes, including any cases where this could be reliably extrapolated, and that missing information is reflected in the denominators in our tables"). I am not convinced that the collection of data from birth reports with poor or inaccurate documentation is valid for drawing the conclusions that the authors have drawn in this work. It is unclear to me how missing data can be extrapolated from documented data points.

Also I lack access to the manuscript by A. Reitter, "K2 Medical Systems Perinatal Training: Intrapartum Breech Management. In: Maternity Crisis Management Training. 2021", which was given as a reference to this data analysis, where the methods of extrapolation of Data is described. Could you please send me an access to this referenced manuscript? To be able to understand the determination of the data points, I need the specified literature.

If data from the birth report are interpreted using invalid data collection or by a nonvalidated method, the statements made from this cannot be made, even if the authors write in the corrected version of their work: "Systematic errors in the sample are likely to have been applicable to both cases and controls, and these results may change if data are collected prospectively".

Even prospective study is mandatory; this paper could have the potential to change the management of vaginal breech delivery by introducing time points and time limits. Therefore time limits should be a result of analyzing validated data.

Therefore, my review cannot approve the paper for indexing at this point.

Kind regards,

Gerhard Bogner

**Is the work clearly and accurately presented and does it cite the current literature?**

Yes

**Is the study design appropriate and is the work technically sound?**

Yes

**Are sufficient details of methods and analysis provided to allow replication by others?**

Yes

**If applicable, is the statistical analysis and its interpretation appropriate?**

Yes

**Are all the source data underlying the results available to ensure full reproducibility?**

Yes

**Are the conclusions drawn adequately supported by the results?**

Yes

***Reviewer Expertise:*** fullfilled

**I confirm that I have read this submission and believe that I have an appropriate level of expertise to state that I do not consider it to be of an acceptable scientific standard, for reasons outlined above.**

**Reviewer Report 20 September 2022**

https://doi.org/10.3310/nihropenres.14441.r28783

**© 2022 Small K.** This is an open access peer review report distributed under the terms of the Creative Commons Attribution License, which permits unrestricted use, distribution, and reproduction in any medium, provided the original work is properly cited.

✔ **Kirsten Small**^iD^

Transforming Maternity Care Collaborative, School of Nursing and Midwifery, Logan Campus, Griffith University, Meadowbrook, QLD, Australia

Thank you for your additional work on your paper. The logic line of the argument is well structured and appropriate caution has been applied when interpreting the significance of the findings. My previous recommendations for the paper have been addressed in this revised version.

**Is the work clearly and accurately presented and does it cite the current literature?**

Yes

**Is the study design appropriate and is the work technically sound?**

Yes

**Are sufficient details of methods and analysis provided to allow replication by others?**

Yes

**If applicable, is the statistical analysis and its interpretation appropriate?**

Yes

**Are all the source data underlying the results available to ensure full reproducibility?**

Yes

**Are the conclusions drawn adequately supported by the results?**

Yes

***Reviewer Expertise:*** Clinical expertise in physiological vaginal breech birth, doctorally qualified obstetrician. Intrapartum fetal heart rate monitoring.

**I confirm that I have read this submission and believe that I have an appropriate level of expertise to confirm that it is of an acceptable scientific standard.**

**Version 1**

**Reviewer Report 16 August 2022**

https://doi.org/10.3310/nihropenres.14421.r28736

**© 2022 Small K.** This is an open access peer review report distributed under the terms of the **Creative Commons Attribution License**, which permits unrestricted use, distribution, and reproduction in any medium, provided the original work is properly cited.

？ **Kirsten Small**^iD^

Transforming Maternity Care Collaborative, School of Nursing and Midwifery, Logan Campus, Griffith University, Meadowbrook, QLD, Australia

Congratulations on adding new and useful information to our understanding of how best to care for women during their vaginal breech birth. The writing is clear, logical, and free from errors. The abstract is well structured and reflects the key elements of the study. Key literature regarding vaginal breech birth is addressed in the introduction. The methods used were explained well. The findings are presented in an easy to understand manner. The discussion provides context for the findings, demonstrating a detailed knowledge of the field. The discussion is also used to further explore and theorise some of the unexpected findings of the study.

I have some suggestions for how I believe you can further strengthen the paper:

○ You use the abbreviation VBB once in the introduction without having defined it, but then use vaginal breech birth for the remainder of the paper. Please edit this to achieve consistency throughout.
○ You explain that the Algorithm was not in use at the site during the period when the births in this study occurred. It would be useful to have additional contextual information about whether other approaches to providing training for maternity staff in vaginal breech birth had been undertaken, and what policy guidance was in place at the site at the time.
○ Can you please comment on whether there were challenges in ascertaining all the variables of interest? Was it standard practice to document the timing of each stage of emergence? Were there instances where data were missing and if so, how was this handled?
○ I would appreciate further information about why admission to the nursery, and not another measure of morbidity, was chosen as the primary end point (in the absence of mortality data). It would also be of use to include further data about why cases were admitted, for how long, and whether there were morbidities related to the mode of birth and / or the techniques used to facilitate birth.
○ Admission to the nursery is a clinical decision and therefore subjective. Given that cord blood gas results were better among cases, and not all cases appeared to have abnormal Apgar scores at 5 minutes of age, the reader is left wondering what prompted the decision for admission. A possible alternate explanation of your findings is that neonatal staff elected to admit infants after vaginal breech births that appeared to have been difficult or prolonged as a matter of precaution, rather than because of suspected or actual harm. It would strengthen your argument that delay was associated with poorer outcomes, if you were to show that there were indeed worse outcomes among those admitted to the nursery. At present, in the absence of this information, I think that the absence of additional evidence of harm due to delay should be acknowledged as a limitation of the study. Hopefully, you can provide additional data that will dispel this concern.
○ In table 1 you refer to "intermittent monitoring". Do you mean intermittent auscultation / fetal heart rate monitoring? There are many forms of monitoring in labour so the term here is unclear. I would presume that during emergence that at least one maternity professional was present and constantly monitoring the overall situation.
○ In the discussion, paragraph two begins with a suggestion to take a "more active" approach - which begs the question, compared to what? Simply stating that an active approach is supported would be as impactful, given that the algorithm provides clarity on when and what activity is advised.
○ In the second paragraph of the interpretation section, you appear to say that a 13 minute delay after the birth of the pelvis is the same as head entrapment, and that this is what so many clinicians fear. Can you rephrase this sentence to be sure you are clearly expressing the point that was intended here? I believe the learning point (that inaction early in emergence contributes to poor tone and therefore difficulty in birthing the head) is an important one and it is slightly lost in the current wording of the sentence.
○ You have acknowledged the limitations of using a retrospective case-control methodology. I do believe that the knowledge generated from this small retrospective study remains useful. Previous recommendations for the timing of intervention in vaginal breech birth guidelines were based on less evidence than this. You make it clear that further evaluation of the Algorithm is underway within the Optibirth randomised controlled trial. Prospective evaluation of the Algorithm will overcome the limitations inherent in this type of methodology.
○ At present, your conclusion reflects a broad understanding of the potential for the Algorithm. I would like to see the conclusion instead focus more clearly on providing a summary of the findings of this particular piece of research and the implications for practice, education, and /or future research.

Thank you for the opportunity to review the paper.

**Is the work clearly and accurately presented and does it cite the current literature?**

Yes

**Is the study design appropriate and is the work technically sound?**

Yes

**Are sufficient details of methods and analysis provided to allow replication by others?**

Yes

**If applicable, is the statistical analysis and its interpretation appropriate?**

Yes

**Are all the source data underlying the results available to ensure full reproducibility?**

Yes

**Are the conclusions drawn adequately supported by the results?**

Yes

***Reviewer Expertise:*** Clinical expertise in physiological vaginal breech birth, doctorally qualified obstetrician. Intrapartum fetal heart rate monitoring.

**I confirm that I have read this submission and believe that I have an appropriate level of expertise to confirm that it is of an acceptable scientific standard, however I have significant reservations, as outlined above.**

**Author Response 03 Sep 2022**
**Shawn Walker,** King's College London, 10th floor North Wing, St Thomas' Hospital, UK We thank the reviewer for their kind words and considered review, which we agree will strengthen the work. We have replaced the single incidence of the abbreviation VBB with the complete phrase, for consistency. We have added a brief paragraph about the training and guidance in use at the site during the study in our methods section. This is brief because it is not possible to determine which staff had exposure to which training, whether they attended additional training through other organisations, whether they had undertaken mandatory training, what was included in that training, etc. While we agree this would be interesting, and are conducting a prospective study where these elements are accounted for, our purpose in this study was to look in detail at births in a standard practice environment to test a specific theory about how birth intervals relate to neonatal admissions or deaths. ***Can you please comment on whether there were challenges in ascertaining the variables of interest? Was it standard practice to document the timing of each stage of emergence? Were there instances where data were missing and if so, how was this handled?*** - Yes, there were many challenges. However, it is standard practice during obstetric emergencies in the UK to appoint a scribe who writes down what is happening with as much detail as possible. This is easier in births where the emergence is slower. Documentation of timing of cord clamping and cutting has been an auditable criteria in the UK since 2014, when NICE guidance recommended delayed cord clamping. Where data was missing and could not be reliably inferred from available documentation, it was not included. This is reflected in the denominators we report in our tables. Data points were only extrapolated where other reliable parameters were documented. We have now included more information about this in our methods section. ***I would appreciate further information about why admission to the nursery, and not another measure of morbidity, was chosen as the primary end point (in absence of mortality data). It would also be of use to include further data about why cases where admitted, for how long, and whether there were morbidities related to the mode of birth and/or the techniques used to facilitate the birth.*** - Admission to neonatal intensive care was a pre-specified endpoint, along with neonatal death. We chose neonatal admission for 3 reasons: 1) it happens more often than severe adverse outcomes; 2) it has economic implications, even if admission is due to practitioner caution rather than true morbidity; and 3) it is an important outcome for service users. We have a very active Patient and Public Involvement group that informs our programme of research, and they have repeatedly told us reducing separation of mother and baby at birth is important to them. We have included further information in the results and discussion about the documented reason for admission, which was in all cases documented as the need for resuscitation. We have not collected information about length of admission in this study, although we are collecting that information in our prospective work. **Re: admission to the nursery is a clinical decision and therefore subjective…**: We agree with this. However, we would argue that, in the eyes of service users, separation from the newborn around the time of birth is a harm, even though it is not considered a serious adverse outcome by healthcare scientists. Whether this results from practitioner nervousness or actual morbidity requiring higher-level care, the separation itself is an important consequence. While practitioners may judge a successful resuscitation followed by an overnight admission for observation, with no long-term sequelae, to be a 'good outcome,' service users would prefer to avoid this if possible. While we have not gathered and cannot report additional evidence of serious harm due to delay, the lack of admission following births which had on average earlier intervention does provide some evidence that the earlier intervention, in this context, is not resulting in additional harm. We have expanded on this in our limitations section. ***Table 1, "intermittent monitoring":*** We have changed this for clarity. All births were attended, as this was an inclusion criteria, but not all occurred in an obstetric unit. In the UK, women with a known breech presentation are offered continuous electronic fetal heart monitoring, but some decline this. Over half of the sample included births where breech presentation was first diagnosed in labour, and depending on the timing of diagnosis, there may not have been time to offer continuous electronic fetal heart rate monitoring or to transfer to an obstetric unit. ***At present, your conclusion reflects a broad understanding of the potential for the Algorithm. I would like to see the conclusion instead focus more clearly on providing a summary of the findings of this particular piece of research and the implications for practice, education, and/or future research***. We have revised our conclusion as suggested, thank you. ***Competing Interests:*** No competing interests were disclosed.

**Reviewer Report 15 August 2022**

https://doi.org/10.3310/nihropenres.14421.r28738

**© 2022 Bogner G.** This is an open access peer review report distributed under the terms of the Creative Commons Attribution License, which permits unrestricted use, distribution, and reproduction in any medium, provided the original work is properly cited.

✖ **Gerhard Bogner**^iD^

Department of Obstetrics and Gynaecology, Paracelsus Medical University, Salzburg, Austria

The study aims to test a self-designed algorithm for the course of a vaginal breech delivery. In principle, it is a high goal to describe parameters that are associated with a poorer neonatal outcome, record them and prevent them through obstetric intervention. It is questionable to me whether the given study design answers the question of the role of the timing of birth and its impact on the perinatal outcome:

1. Retrospective data collection on a large cohort of births from large hospitals that do not appear to correspond to the hospitals where the authors work.
2. Data collection from a retrospective collective is always imprecise and mostly incorrect (missing documentation of interventions and times).
3. It is not possible to evaluate measures out of routine collection and documentation of timepoints of fetal pelvic entry, rumping, the emergence of the umbilical cord insertion, as well as timing of umbilical cord clamping and time to delivery are routinely not recorded in the birth report.
4. Even recording the birth phase and pressing phase is subject to inaccuracies.
5. As long as these times are only indirectly estimated with unclear methodology, conclusions about the calculated periods are implausible (I do not have access to the specified literature on the calculation of these parameters as indicated, "Maternity Crisis Management Training 2021").
6. Tools, that were developed to estimate timepoints of delivery out of an imprecise or incomplete report have to be validated.
7. Only a study with a prospective design and study protocol can accomplish this task.

Some results are open or questionable:

1. Why are the pH-values in the tested group (=cases with 100% transfer rate ad NICU) higher than in the comparison group (=controls)? Early umbilical cord clamping as an argument is not plausible. Is the transfer to NICU due to reduced APGAR a result of manual intervention?
2. Is a parameter to detect a higher neonatal transfer rate ad NICU with sensitivity of 66% a good parameter to recommend consequencies in delivery management? Is the collective of 15 cases perhaps too small?
3. What were the actual reasons for transferring the children to the NICU (immaturity? infection? hypoglycemia? etc.)
4. In how many births could the necessary data not be read out from the documentation, or could it be read incompletely or with uncertainty?
5. Rapid, simple vaginal birth always tends to have a better outcome for the children, while protracted birth leads to a poorer outcome, with and without intervention. What weighs more heavily for the newborn poor outcome from intervention and/or acidosis?

As long as the collection of the data at the calculated times (timeline) is not comprehensible, this study cannot be approved for indexing in this form

**Is the work clearly and accurately presented and does it cite the current literature?**

Partly

**Is the study design appropriate and is the work technically sound?**

Partly

**Are sufficient details of methods and analysis provided to allow replication by others?**

No

**If applicable, is the statistical analysis and its interpretation appropriate?**

I cannot comment. A qualified statistician is required.

**Are all the source data underlying the results available to ensure full reproducibility?**

Partly

**Are the conclusions drawn adequately supported by the results?**

No

***Reviewer Expertise:*** fullfilled

**I confirm that I have read this submission and believe that I have an appropriate level of expertise to state that I do not consider it to be of an acceptable scientific standard, for reasons outlined above.**

**Author Response 03 Sep 2022**
**Shawn Walker,** King's College London, 10th floor North Wing, St Thomas' Hospital, UK We thank Reviewer #1 for the time spent reviewing this work with rigour. Reviewer #1 has expressed concerns with the design of the research, and on this basis has recommended that the research not be indexed. The NIHR Open Research category, "Not approved," indicates: Crucial substantial revisions will be required for the paper to pass peer review. Some of the criticisms concern aspects of the design that cannot be changed. Therefore, we have responded to the criticisms with a view to enabling the research to be indexed, with awareness that the reviewer's comments will remain publicly available alongside it. We are committed to transparency and feel it is important that concerns like these are available to those who might consider using our research. These are our responses to the specific criticisms.

1. ***Retrospective data collection on a large cohort of births from large hospitals that do not appear to correspond to the hospitals where the authors work.*** - We do not understand why a large cohort, a large hospital, or the fact that the authors did not work at the hospital during the period when the births took place would undermine the research. We purposively sought a sample from a hospital where we did not work clinically. We have acknowledged in our limitations discussion that the research is retrospective and that prospective research would be better, and we have clarified that this small study was a precursor to a larger prospective study. The prospective research is in progress. Sharing this retrospective work explains the clinical recommendations we are testing in the prospective work and the hypotheses on which they are based.
2. ***Data collection from a retrospective collective is always imprecise and mostly incorrect (missing documentation of interventions and times).*** - This is true, and we have acknowledged this as a limitation in our discussion. We have transparently reported the number of cases where we were able to collect this information because it was documented as denominators in each of our tables. It is common practice in UK maternity care, and a part of training for these events, to appoint a 'scribe' during any obstetric emergency, who documents times and interventions as accurately as possible. In addition, whenever a baby is admitted to the neonatal unit, a risk review is conducted, which includes a reconstruction of a critical timeline as accurately as possible. This would then be included in the person's notes that were reviewed to obtain this sort of information.
3. ***It is not possible to evaluate measures out of routine collection and documentation of timepoints of fetal pelvic entry, rumping, the emergence of the umbilical cord insertion, as well as timing of umbilical cord clamping and time to deliver are routinely not recorded in the birth report.*** - As we have answered above, we have reported transparently how often these were reported and could be included in our analysis, in each of our tables. Some practitioners at this site participated in training based on the Algorithm in 2018, and this may have influenced their awareness of the importance of recording these data points. Timing of umbilical cord clamping has been routinely recorded in the UK, since 2014 when NICE guidance recommended delayed cord clamping and listed it as an auditable criteria. Many other research studies have been done around timing of umbilical cord clamping. Recommendations on breech delivery timings are routinely made in obstetric textbooks. A key motivator for this study was that these recommendations are based on a lack of evidence at best, and some may be actively harmful. It is difficult to determine how to test these beliefs and develop a more robust evidence base without making some attempt to measure what these timings are in actual births.
4. ***Even recording the birth phase and pressing phase is subject to inaccuracies.*** - This is true. However, many obstetric midwifery guidelines do provide guidance on normal ranges for how long labour or pushing lasts and when it should be considered abnormal, when intervention is indicated. Almost all of the studies that underpin these recommendations are based on retrospective data collection, which is commonly understood to be less accurate than prospective data but the best we have to start with initially.
5. ***As long as these times are only indirectly estimated with unclear methodology, conclusions about the calculated periods are implausible.*** - We have reported transparently how often we were able to extract a time for each event from the maternity notes to include in our analysis. We used a structured data collection tool when reviewing the notes to extract this information. We have been clear in discussing the study limitations that these findings will need to be tested in further studies with a larger sample and prospective data collections, but we argue that the current study findings are of sufficient interest to disseminate on this basis.
6. ***Tools that were developed to estimate timepoints of delivery out of an imprecise or incomplete report have to be validated.*** -The tool is not designed to and was not used to estimate timepoints. It is a data collection tool. We extracted the data from the notes where they were reported. Our tool specified the data items that were extracted, which were also pre-specified in the protocol. We acknowledge that sometimes providers documenting care may have estimated timepoints when documenting in retrospect, but this would have been as accurate as any other maternity documentation.
7. ***Only a study with a prospective design and study protocol can accomplish this task.*** - This study provided important groundwork for a prospective study, trialling the use of the tool and establishing the value of a larger scale test of the hypotheses provided in the current study. As noted above, the study limitations were discussed and a prospective study is now in progress. However, there are inherent challenges in developing and conducting a prospective study of this topic - vaginal breech birth is now uncommon and the rate of adverse events implies an unfeasibly long time frame or involvement of a large number of services. In addition, the resources required to ensure fully accurate and complete prospective recording are considerable. These research challenges and the current state of lack of any formal evidence basis for current practice recommendations, in our view, demonstrate the value of sharing the findings of this study.

Some results are open or questionable:

1. ***Why are the pH-values in the tested group (=cases with 100% transfer rate ad NICU) higher than in the comparison group (=controls)? Early umbilical cord clamping as an argument is not plausible. Is the transfer to NICU due to reduced APGAR a result of manual intervention?*** - We also found this outcome surprising, leading us to have several conversations with experts who have conducted research on the timing of umbilical cord clamping and its relationship to cord gases. We intend to do further research in this area and have included it as an important data point in our prospective study, as it is also very important to service users. The questions arising from these results have also been discussed in the article.
  All neonatal admissions were following neonatal resuscitation, and this was documented as the reason for neonatal admission. This has now been clarified in the report of results. Our data indicated that manual interventions were initiated later on average among births where transfers to the NICU took place. In other reports of neonatal poor outcomes, such as the Term Breech Trial (https://www.thelancet.com/journals/lancet/article/PIIS0140-6736(00)02840-3/fulltext ), complications of hypoxia outnumber complications of trauma. Therefore, we do not consider our data to indicate that earlier intervention, with appropriate technique, necessarily results in complications causing NICU admission. Concerns about trauma may be greater in other settings using different methods of delivery, and we intend to investigate this further in our on-going work.
2. ***Is a parameter to detect a higher neonatal transfer rate ad NICU with a sensitivity of 66% a good parameter to recommend consequences in delivery management? Is the collective of 15 cases perhaps too small?*** - Our recommendation pre-dates this study and came from a study of 42 videos (https://onlinelibrary.wiley.com/doi/full/10.1111/birt.12480), in which the timings were recorded accurately, with precision. The purpose of this study was to test whether the recommendations in the Algorithm would help predict NICU admission. They did, but they more accurately predicted no NICU admission (the true negative rate).
  It is important that the reviewer's dissent to this recommendation remain a part of the public record of this research. This dissent is consistent with the majority opinion in obstetrics, which has historically and in recent history suggested that much longer intervals are 'safe' in vaginal breech births and that intervention causes more injury than it prevents. It is also important that the basis for our recommendations are a matter of public record. We are testing them with a further multi-centre study and collecting the same data points in the OptiBreech Trial. If we are wrong, and prospective data collection is inconsistent with our findings or recommendations, this will soon become apparent.
3. ***What were the actual reasons for transferring the children to the NICU (immaturity? infection? hypoglycemia? Etc.)*** - We did not collect this information in this study and therefore cannot report it. All births occurred after 37 weeks; therefore immaturity was not a cause of admission. All neonatal admissions reported occurred immediately following birth; therefore hypoglycemia is unlikely to be a significant cause, as it is later in the neonatal period.
4. ***In how many births could the necessary data not be read out from the documentation, or could it be read incompletely or with uncertainty?*** - We have reported our denominators (the available data points) transparently in our tables. We only included parameters which were clearly documented or could be reliably inferred. For example, in a number of births the birth of the pelvis and the birth of the head occurred within the same minute, in which case the data collector considered it reliable to assume that the birth of the umbilicus occurred within the same minute.
5. ***Rapid, simple vaginal birth always tends to have a better outcome for the children, while protracted birth leads to a poorer outcome, with and without intervention. What weighs more heavily for the newborn poor outcome from intervention and/or acidosis?*** - We agree with the first statement, although our experience and the evidence in this study indicates that intervention can often be rapid, simple and effective at preventing protracted births. In our OptiBreech trial protocol, encouraging maternal movement and effort is the first recommended intervention; in current practice, this is often avoided, and women are instructed instead to, "Breathe and wait for the next contraction." In the Term Breech Trial, complications of asphyxia were more common than traumatic neonatal injuries. In the PREMODA study (https://www.ajog.org/article/S0002-9378(05)02440-3/fulltext), systematic manoeuvres were used routinely in 30.3% of births, with much better outcomes than those reported in the Term Breech Trial. While we are not arguing that every birth requires systematic manoeuvres, we feel that assistance is wrongly avoided in many breech births and that harm results from this avoidance. Our trial protocol also recommends a lower threshold for intervention than current national guidelines in the UK. The evidence in this report partly explains why.
6. ***As long as the collection of the data at the calculated times (timeline) is not comprehensible, this study cannot be approved for indexing in this form.*** - We respectfully ask the reviewer to clarify what would make collection of this data comprehensible. We readily accept that use of a pro forma for documentation purposes, as we are doing in the OptiBreech Trial, has made it somewhat easier. However, documentation of key points, such as when buttocks are visible or born, when legs and arms are born, when the head is born and when the umbilicus is cut, is considered best practice already in UK maternity care.

The reviewer has answered 'no' to the question, "Are sufficient details of methods and analysis provided to allow replication by others?". We understand the concerns were related to how data points were extracted from the notes, and we have provided clarification around this in our description of methods. We are replicating the study at multiple sites following a similar protocol and welcome others to do the same. Others may find different results or interpret them differently. We encourage replication and publication of this topic. If we are incorrect, and earlier intervention causes harm, it is important that this be apparent as soon as possible. If we are correct, and babies would benefit from earlier intervention to prevent unnecessary hypoxia, further evidence will clearly be needed to achieve a change in practice. ***Competing Interests:*** No competing interests were disclosed.
