# Supplementary material for: Optimal time intervals for vaginal breech births: a case-control study
Source: NIHR Open Res. 2022 Sep 16;2:45. Originally published 2022 Aug 4. [Version 2] doi: 10.3310/nihropenres.13297.2 (PMC7614205; doi:10.3310/nihropenres.13297.2)
Supplement: Peer review history

# Reviewer report: Reviewer response for version 2

**DOI:** 10.3310/nihropenres.14441.r28863

**Published:** 2023-01-11

**Authors:** Rachna Bahl

**Affiliations:** 1 Department of Obstetrics and Gynaecology, St. Michael's Hospital, University of Bristol, Bristol, UK

This is an important study that challenges the traditional teaching of breech birth. This is also the first study of this kind.

1. My main concern is the strength of recommendation in paragraph 3 of the discussion section. That its based on a review of 12 cases of breech births leading to NICU admissions. The data is retrospective and the notes are often written after the birth and maybe influenced by the outcome. The number of breech births is small overall. I would suggest rephrasing the paragraph.
2. I am aware that there is a highly skilled team of midwives performing these births. It would be interesting to know the experience of midwives conducting the births in this cohort. Often in obstetrics the skill of person making decisions and performing the action is very important to the outcome.
3. The authors have justified not taking parity and the birth weight into account. Whilst I agree with their reasons, I do believe that a difference in parity needs to be excluded.
4. Can the authors provide information on duration of NICU stay and the final diagnosis? Also were there babies from the control group who were subsequently admitted to NICU? This is important given worse pH in the control group.
5. The conclusion of the paper talks about a 3 min interval between birth of pelvis and head. The protocol says 5 min. Kindly adhere to the timings in the protocol and the results section of the abstract throughout the manuscript.
6. Table 2 gives units for cord bloods and Apgars as minutes. Please amend.

Is the work clearly and accurately presented and does it cite the current literature?

Yes

If applicable, is the statistical analysis and its interpretation appropriate?

I cannot comment. A qualified statistician is required.

Are all the source data underlying the results available to ensure full reproducibility?

Partly

Is the study design appropriate and is the work technically sound?

Partly

Are the conclusions drawn adequately supported by the results?

Yes

Are sufficient details of methods and analysis provided to allow replication by others?

Yes

Reviewer Expertise:

Intrapartum care

I confirm that I have read this submission and believe that I have an appropriate level of expertise to confirm that it is of an acceptable scientific standard, however I have significant reservations, as outlined above.

# Reviewer report: Reviewer response for version 2

**DOI:** 10.3310/nihropenres.14441.r28865

**Published:** 2022-12-20

**Authors:** Marit L. Bovbjerg

**Affiliations:** 1 Epidemiology Program, College of Public Health and Human Sciences, Oregon State University, Corvallis, OR, USA

This is a case control study designed to assess timing standards and protocols for vaginal breech birth, in terms of reducing NICU admissions. I applaud the authors for publishing this manuscript open access; vaginal breech is a common enough occurrence that we should know how to do it well. The sample size is very small, and as currently written some of the methods are either not well explained or not exactly correct. The manuscript is well-written.

 Abstract

- Please clarify who are ‘cases’ and ‘controls’. I started with the abstract, and assumed cases would be neonates who experienced birth related hypoxia. But no, from the methods section, they’re kids who were in the NICU or dead. The latter two of which are great patient-centered outcomes! Suggest (here and in the intro) therefore not starting the whole thing out with “hypoxic injury” then--it seems to the reader like that will be the main issue. But no. The main issue is seriously brain damaged/dead babies. Start there, and then say “secondary to hypoxia during birth” at some point.

Introduction

- Well written. Clear. Consise.
- However, as I say above under ‘abstract’ suggest not leading off with hypoxia, since that’s not really the issue. That’s a mediator, on the road to brain damage/death.
- my only other suggestion is to strengthen the objective statement and actually say what you’re doing. Instead of “we hoped to” say “our objective was to”
- And please make it SPECIFIC! “seeing whether this thing we published previously is correct” is not specific. Seeing whether a given cutoff is the best one is more specific. Defining optimal times for intervention is specific. etc.

Methods

- Thanks for including a power calculation! A bigger sample would have been better, of course, bc those confidence intervals in table 1 are awfully wide, and many of them cross 1. More clarity could come with a bigger sample; but I understand you are using this algorithm for an upcoming larger trial, so perhaps you can repeat this analysis with that larger sample.
- Please clarify sampling procedure. If what you did is
  - take all the vaginal breech births
    - but starting from when in labor? All who were in second stage with a breech fetus? So planned vaginal breech who head for cesarean during first stage are out? Or something else? What are that hospital’s protocols for breech? Who can plan one? Who heads straight for the OR with unplanned (eg, I assume footling/knee are out…also primips?)
  - of those vaginal breech births, find the first 15 you could who ended up in the NICU (or dead, but there weren’t any of those)
    - were there the a priori time frames on those? eg, NICU within the first 24 hours? Dead in the first week?
  - then took the previous two vaginal breeches that did NOT result in a case
    - but what if they did? Like, what if there had been a breech/NICU, then the previous vaginal breech was ALSO a NICU? Then who would the controls have been? I’m guessing it didn’t come up, but what was your plan if it had?
- Please clarify what a “ROC curve analysis” is. You drew one, then….used Youden’s index? calculated the area under the curve? (in which case, why do logistic regressions?)
- Given extremely small sample size, logistic regressions aren’t really OK with more than 2-3 dichotomous covariables. That will overfit the models, and make them less likely to apply to additional/external samples.
- I don’t understand what you did with logistic regressions. If it’s “predictive values of meeting or exceeding the time limit”…so then the outcome of your logistic regression is NICU/death, and the exposure is “>= time limit”, and you threw in a couple potential confounders? That’s not really a “predictive value” (which since you’re also doing ROC curves, PPV and NPV are things related to sensitivity and specificity)

Results

- Is PAC just the area under the ROC curve?
- Table 1 - why are there asterisks for three of the outcomes? I first assumed bc there were 0 cells in the 2x2 table, but no, there were plenty of events?
- The ROC curve--ah. I’m not sure this is useful, given each “test” had only one possible cutpoint. Usually ROC curves are for comparing multiple possible cutpoints (eg, rumping to birth 5 min, 6 min, 7 min, 8 min, 9 min), and/or for area under the curve, which is overall discriminatory power, but again interpretation assumes multiple possible cutpoints.
- Please discuss PPV and NPV separately from sens/spec, since the former will vary based on prevalence of NICU admission in a given sample
- Given opening paragraph, I’m not sure what you did, methods-wise. Put all three timepoints (rumping, pelvis, umbilicus) into the model, as independent predictors, each as a yes/no variable given the 3/5/7 cutoffs? OK. So no confounders? (good, bc your sample is too small)
- Second paragraph of results…wait, so you did test multiple possible cutpoints? Here it mentions >3 min for pelvis and >3 for umbilicus? Please clarify, the logistic regressions and the ROC curve analyses
- Table 2 - did all cases have interventions? If not, please differentiate in the table. If yes, please make that as a separate point.
- Towards the end, the parts about how long before interventions--that needs to be mentioned more in the methods. There’s just the one sentence about calculating time to event intervals. Also, please say calculate time to interventions, and then we compared time to interventions by..
- A time-line-y figure with times to interventions, say, with different colors for cases and controls, would really make your point about intervening earlier

Discussion

- Given that I don’t think you explored other time limits, you can’t say it’s “normal” for THIS to happen in under THAT amount of time. It might be better at 6 min 30 sec, say. (Also suggest avoiding “normal” as a word to mean anything other than it’s statistical definition)
- If your first sentence under strengths is about delays in intervening…then maybe that’s the overall study objective?
- Second paragraph in interpretation--Your data doesn't say anything about poor tone/deflection, nor 13 minutes for head birth…that I saw? If this is referencing something else, please cite. Otherwise, please don’t speculate beyond your data.
- I like your critique of current thinking (hands off, let it hang, etc)--we should be using data!

Minor points:

- Second paragraph of the introduction, last sentence--do you mean birthweights greater than the 90 ^th^ percentile? I thought it was macrosomic babies who didn’t do so hot with vaginal breech. Not the little ones.
- “rumping” in lieu of “crowning” in your algorithm cracks me up
- “BAME” is absolutely not how we would write about minoritized people in the USA, but if it’s a standard UK term then fine
- Consider not identifying the exact hospital, especially given you have a study sample explicitly selected bc of poor outcomes. people might be identifiable, if they’ve told their friends details, for instance. (wait, there’s only NICUs, no deaths. Not as bad, then. But still…consider just saying “a large urban hospital in the UK with 5000 births per year”)

Is the work clearly and accurately presented and does it cite the current literature?

Partly

If applicable, is the statistical analysis and its interpretation appropriate?

Partly

Are all the source data underlying the results available to ensure full reproducibility?

No

Is the study design appropriate and is the work technically sound?

Partly

Are the conclusions drawn adequately supported by the results?

Yes

Are sufficient details of methods and analysis provided to allow replication by others?

No

Reviewer Expertise:

perinatal epidemiology; maternity care health services research

I confirm that I have read this submission and believe that I have an appropriate level of expertise to confirm that it is of an acceptable scientific standard, however I have significant reservations, as outlined above.

# Reviewer report: Reviewer response for version 2

**DOI:** 10.3310/nihropenres.14441.r28784

**Published:** 2022-10-31

**Authors:** Gerhard Bogner

**Affiliations:** 1 Department of Obstetrics and Gynaecology, Paracelsus Medical University, Salzburg, Austria

Unfortunately, I am not yet convinced by the author's answer ("Clarification is provided about how data was extracted from the notes, including any cases where this could be reliably extrapolated, and that missing information is reflected in the denominators in our tables"). I am not convinced that the collection of data from birth reports with poor or inaccurate documentation is valid for drawing the conclusions that the authors have drawn in this work.

 It is unclear to me how missing data can be extrapolated from documented data points.

 Also I lack access to the manuscript by A. Reitter, "K2 Medical Systems Perinatal Training: Intrapartum Breech Management. In: Maternity Crisis Management Training. 2021", which was given as a reference to this data analysis, where the methods of extrapolation of Data is described. Could you please send me an access to this referenced manuscript? To be able to understand the determination of the data points, I need the specified literature.

 If data from the birth report are interpreted using invalid data collection or by a nonvalidated method, the statements made from this cannot be made, even if the authors write in the corrected version of their work: "Systematic errors in the sample are likely to have been applicable to both cases and controls, and these results may change if data are collected prospectively".

 Even prospective study is mandatory; this paper could have the potential to change the management of vaginal breech delivery by introducing time points and time limits. Therefore time limits should be a result of analyzing validated data. 

 Therefore, my review cannot approve the paper for indexing at this point.

 Kind regards,

 Gerhard Bogner

Is the work clearly and accurately presented and does it cite the current literature?

Partly

If applicable, is the statistical analysis and its interpretation appropriate?

I cannot comment. A qualified statistician is required.

Are all the source data underlying the results available to ensure full reproducibility?

Partly

Is the study design appropriate and is the work technically sound?

Partly

Are the conclusions drawn adequately supported by the results?

No

Are sufficient details of methods and analysis provided to allow replication by others?

No

Reviewer Expertise:

fullfilled

I confirm that I have read this submission and believe that I have an appropriate level of expertise to state that I do not consider it to be of an acceptable scientific standard, for reasons outlined above.

# Reviewer report: Reviewer response for version 2

**DOI:** 10.3310/nihropenres.14441.r28783

**Published:** 2022-09-20

**Authors:** Kirsten Small

**Affiliations:** 1 Transforming Maternity Care Collaborative, School of Nursing and Midwifery, Logan Campus, Griffith University, Meadowbrook, QLD, Australia

Thank you for your additional work on your paper. The logic line of the argument is well structured and appropriate caution has been applied when interpreting the significance of the findings. My previous recommendations for the paper have been addressed in this revised version.

Is the work clearly and accurately presented and does it cite the current literature?

Yes

If applicable, is the statistical analysis and its interpretation appropriate?

Yes

Are all the source data underlying the results available to ensure full reproducibility?

Yes

Is the study design appropriate and is the work technically sound?

Yes

Are the conclusions drawn adequately supported by the results?

Yes

Are sufficient details of methods and analysis provided to allow replication by others?

Yes

Reviewer Expertise:

Clinical expertise in physiological vaginal breech birth, doctorally qualified obstetrician. Intrapartum fetal heart rate monitoring.

I confirm that I have read this submission and believe that I have an appropriate level of expertise to confirm that it is of an acceptable scientific standard.

# Reviewer report: Reviewer response for version 1

**DOI:** 10.3310/nihropenres.14421.r28736

**Published:** 2022-08-16

**Authors:** Kirsten Small

**Affiliations:** 1 Transforming Maternity Care Collaborative, School of Nursing and Midwifery, Logan Campus, Griffith University, Meadowbrook, QLD, Australia

Congratulations on adding new and useful information to our understanding of how best to care for women during their vaginal breech birth. The writing is clear, logical, and free from errors. The abstract is well structured and reflects the key elements of the study. Key literature regarding vaginal breech birth is addressed in the introduction. The methods used were explained well. The findings are presented in an easy to understand manner. The discussion provides context for the findings, demonstrating a detailed knowledge of the field. The discussion is also used to further explore and theorise some of the unexpected findings of the study. 

 I have some suggestions for how I believe you can further strengthen the paper:

- You use the abbreviation VBB once in the introduction without having defined it, but then use vaginal breech birth for the remainder of the paper. Please edit this to achieve consistency throughout. 
- You explain that the Algorithm was not in use at the site during the period when the births in this study occurred. It would be useful to have additional contextual information about whether other approaches to providing training for maternity staff in vaginal breech birth had been undertaken, and what policy guidance was in place at the site at the time. 
- Can you please comment on whether there were challenges in ascertaining all the variables of interest? Was it standard practice to document the timing of each stage of emergence? Were there instances where data were missing and if so, how was this handled? 
- I would appreciate further information about why admission to the nursery, and not another measure of morbidity, was chosen as the primary end point (in the absence of mortality data). It would also be of use to include further data about why cases were admitted, for how long, and whether there were morbidities related to the mode of birth and / or the techniques used to facilitate birth. 
- Admission to the nursery is a clinical decision and therefore subjective. Given that cord blood gas results were better among cases, and not all cases appeared to have abnormal Apgar scores at 5 minutes of age, the reader is left wondering what prompted the decision for admission. A possible alternate explanation of your findings is that neonatal staff elected to admit infants after vaginal breech births that appeared to have been difficult or prolonged as a matter of precaution, rather than because of suspected or actual harm. It would strengthen your argument that delay was associated with poorer outcomes, if you were to show that there were indeed worse outcomes among those admitted to the nursery. At present, in the absence of this information, I think that the absence of additional evidence of harm due to delay should be acknowledged as a limitation of the study. Hopefully, you can provide additional data that will dispel this concern. 
- In table 1 you refer to "intermittent monitoring". Do you mean intermittent auscultation / fetal heart rate monitoring? There are many forms of monitoring in labour so the term here is unclear. I would presume that during emergence that at least one maternity professional was present and constantly monitoring the overall situation.  
- In the discussion, paragraph two begins with a suggestion to take a "more active" approach - which begs the question, compared to what? Simply stating that an active approach is supported would be as impactful, given that the algorithm provides clarity on when and what activity is advised. 
- In the second paragraph of the interpretation section, you appear to say that a 13 minute delay after the birth of the pelvis is the same as head entrapment, and that this is what so many clinicians fear. Can you rephrase this sentence to be sure you are clearly expressing the point that was intended here? I believe the learning point (that inaction early in emergence contributes to poor tone and therefore difficulty in birthing the head) is an important one and it is slightly lost in the current wording of the sentence. 
- You have acknowledged the limitations of using a retrospective case-control methodology. I do believe that the knowledge generated from this small retrospective study remains useful. Previous recommendations for the timing of intervention in vaginal breech birth guidelines were based on less evidence than this. You make it clear that further evaluation of the Algorithm is underway within the Optibirth randomised controlled trial. Prospective evaluation of the Algorithm will overcome the limitations inherent in this type of methodology.
- At present, your conclusion reflects a broad understanding of the potential for the Algorithm. I would like to see the conclusion instead focus more clearly on providing a  summary of the findings of this particular piece of research and the implications for practice, education, and /or future research. 

Thank you for the opportunity to review the paper.

Is the work clearly and accurately presented and does it cite the current literature?

Yes

If applicable, is the statistical analysis and its interpretation appropriate?

Yes

Are all the source data underlying the results available to ensure full reproducibility?

Yes

Is the study design appropriate and is the work technically sound?

Yes

Are the conclusions drawn adequately supported by the results?

Yes

Are sufficient details of methods and analysis provided to allow replication by others?

Yes

Reviewer Expertise:

Clinical expertise in physiological vaginal breech birth, doctorally qualified obstetrician. Intrapartum fetal heart rate monitoring.

I confirm that I have read this submission and believe that I have an appropriate level of expertise to confirm that it is of an acceptable scientific standard, however I have significant reservations, as outlined above.

# Response

**Published:** 2022-09-03

**Authors:** Shawn Walker

**Affiliations:** King's College London, UK

We thank the reviewer for their kind words and considered review, which we agree will strengthen the work.

 We have replaced the single incidence of the abbreviation VBB with the complete phrase, for consistency.

 We have added a brief paragraph about the training and guidance in use at the site during the study in our methods section. This is brief because it is not possible to determine which staff had exposure to which training, whether they attended additional training through other organisations, whether they had undertaken mandatory training, what was included in that training, etc. While we agree this would be interesting, and are conducting a prospective study where these elements are accounted for, our purpose in this study was to look in detail at births in a standard practice environment to test a specific theory about how birth intervals relate to neonatal admissions or deaths.

***Can you please comment on whether there were challenges in ascertaining the variables of interest? Was it standard practice to document the timing of each stage of emergence? Were there instances where data were missing and if so, how was this handled?*** – Yes, there were many challenges. However, it is standard practice during obstetric emergencies in the UK to appoint a scribe who writes down what is happening with as much detail as possible. This is easier in births where the emergence is slower. Documentation of timing of cord clamping and cutting has been an auditable criteria in the UK since 2014, when NICE guidance recommended delayed cord clamping. Where data was missing and could not be reliably inferred from available documentation, it was not included. This is reflected in the denominators we report in our tables. Data points were only extrapolated where other reliable parameters were documented. We have now included more information about this in our methods section.

***I would appreciate further information about why admission to the nursery, and not another measure of morbidity, was chosen as the primary end point (in absence of mortality data). It would also be of use to include further data about why cases where admitted, for how long, and whether there were morbidities related to the mode of birth and/or the techniques used to facilitate the birth.*** – Admission to neonatal intensive care was a pre-specified endpoint, along with neonatal death. We chose neonatal admission for 3 reasons: 1) it happens more often than severe adverse outcomes; 2) it has economic implications, even if admission is due to practitioner caution rather than true morbidity; and 3) it is an important outcome for service users. We have a very active Patient and Public Involvement group that informs our programme of research, and they have repeatedly told us reducing separation of mother and baby at birth is important to them. We have included further information in the results and discussion about the documented reason for admission, which was in all cases documented as the need for resuscitation. We have not collected information about length of admission in this study, although we are collecting that information in our prospective work.

**Re: admission to the nursery is a clinical decision and therefore subjective … **: We agree with this. However, we would argue that, in the eyes of service users, separation from the newborn around the time of birth is a harm, even though it is not considered a serious adverse outcome by healthcare scientists. Whether this results from practitioner nervousness or actual morbidity requiring higher-level care, the separation itself is an important consequence. While practitioners may judge a successful resuscitation followed by an overnight admission for observation, with no long-term sequelae, to be a ‘good outcome,’ service users would prefer to avoid this if possible. While we have not gathered and cannot report additional evidence of serious harm due to delay, the lack of admission following births which had on average earlier intervention does provide some evidence that the earlier intervention, in this context, is not resulting in additional harm. We have expanded on this in our limitations section.

***Table 1, “intermittent monitoring”:*** We have changed this for clarity. All births were attended, as this was an inclusion criteria, but not all occurred in an obstetric unit. In the UK, women with a known breech presentation are offered continuous electronic fetal heart monitoring, but some decline this. Over half of the sample included births where breech presentation was first diagnosed in labour, and depending on the timing of diagnosis, there may not have been time to offer continuous electronic fetal heart rate monitoring or to transfer to an obstetric unit.

***At present, your conclusion reflects a broad understanding of the potential for the Algorithm. I would like to see the conclusion instead focus more clearly on providing a summary of the findings of this particular piece of research and the implications for practice, education, and/or future research.*** We have revised our conclusion as suggested, thank you.

# Reviewer report: Reviewer response for version 1

**DOI:** 10.3310/nihropenres.14421.r28738

**Published:** 2022-08-15

**Authors:** Gerhard Bogner

**Affiliations:** 1 Department of Obstetrics and Gynaecology, Paracelsus Medical University, Salzburg, Austria

The study aims to test a self-designed algorithm for the course of a vaginal breech delivery. In principle, it is a high goal to describe parameters that are associated with a poorer neonatal outcome, record them and prevent them through obstetric intervention. It is questionable to me whether the given study design answers the question of the role of the timing of birth and its impact on the perinatal outcome:

1. Retrospective data collection on a large cohort of births from large hospitals that do not appear to correspond to the hospitals where the authors work.
2. Data collection from a retrospective collective is always imprecise and mostly incorrect (missing documentation of interventions and times).
3. It is not possible to evaluate measures out of routine collection and documentation of timepoints of fetal pelvic entry, rumping, the emergence of the umbilical cord insertion, as well as timing of umbilical cord clamping and time to delivery are routinely not recorded in the birth report.
4. Even recording the birth phase and pressing phase is subject to inaccuracies.
5. As long as these times are only indirectly estimated with unclear methodology, conclusions about the calculated periods are implausible (I do not have access to the specified literature on the calculation of these parameters as indicated, "Maternity Crisis Management Training 2021").
6. Tools, that were developed to estimate timepoints of delivery out of an imprecise or incomplete report have to be validated.
7. Only a study with a prospective design and study protocol can accomplish this task.

Some results are open or questionable:

1. Why are the pH-values in the tested group (=cases with 100% transfer rate ad NICU) higher than in the comparison group (=controls)? Early umbilical cord clamping as an argument is not plausible. Is the transfer to NICU due to reduced APGAR a result of manual intervention?
2. Is a parameter to detect a higher neonatal transfer rate ad NICU with sensitivity of 66% a good parameter to recommend consequencies in delivery management? Is the collective of 15 cases perhaps too small?
3. What were the actual reasons for transferring the children to the NICU (immaturity? infection? hypoglycemia? etc.)
4. In how many births could the necessary data not be read out from the documentation, or could it be read incompletely or with uncertainty?
5. Rapid, simple vaginal birth always tends to have a better outcome for the children, while protracted birth leads to a poorer outcome, with and without intervention. What weighs more heavily for the newborn poor outcome from intervention and/or acidosis?

As long as the collection of the data at the calculated times (timeline) is not comprehensible, this study cannot be approved for indexing in this form

Is the work clearly and accurately presented and does it cite the current literature?

Partly

If applicable, is the statistical analysis and its interpretation appropriate?

I cannot comment. A qualified statistician is required.

Are all the source data underlying the results available to ensure full reproducibility?

Partly

Is the study design appropriate and is the work technically sound?

Partly

Are the conclusions drawn adequately supported by the results?

No

Are sufficient details of methods and analysis provided to allow replication by others?

No

Reviewer Expertise:

fullfilled

I confirm that I have read this submission and believe that I have an appropriate level of expertise to state that I do not consider it to be of an acceptable scientific standard, for reasons outlined above.

# Response

**Published:** 2022-09-03

**Authors:** Shawn Walker

**Affiliations:** King's College London, UK

We thank Reviewer #1 for the time spent reviewing this work with rigour. Reviewer #1 has expressed concerns with the design of the research, and on this basis has recommended that the research not be indexed. The NIHR Open Research category, “Not approved,” indicates: Crucial substantial revisions will be required for the paper to pass peer review. Some of the criticisms concern aspects of the design that cannot be changed. Therefore, we have responded to the criticisms with a view to enabling the research to be indexed, with awareness that the reviewer’s comments will remain publicly available alongside it. We are committed to transparency and feel it is important that concerns like these are available to those who might consider using our research.

 These are our responses to the specific criticisms.

1. ***Retrospective data collection on a large cohort of births from large hospitals that do not appear to correspond to the hospitals where the authors work.*** – We do not understand why a large cohort, a large hospital, or the fact that the authors did not work at the hospital during the period when the births took place would undermine the research. We purposively sought a sample from a hospital where we did *not *work clinically. We have acknowledged in our limitations discussion that the research is retrospective and that prospective research would be better, and we have clarified that this small study was a precursor to a larger prospective study. The prospective research is in progress. Sharing this retrospective work explains the clinical recommendations we are testing in the prospective work and the hypotheses on which they are based.
2. ***Data collection from a retrospective collective is always imprecise and mostly incorrect (missing documentation of interventions and times).*** – This is true, and we have acknowledged this as a limitation in our discussion. We have transparently reported the number of cases where we were able to collect this information because it was documented as denominators in each of our tables. It is common practice in UK maternity care, and a part of training for these events, to appoint a ‘scribe’ during any obstetric emergency, who documents times and interventions as accurately as possible. In addition, whenever a baby is admitted to the neonatal unit, a risk review is conducted, which includes a reconstruction of a critical timeline as accurately as possible. This would then be included in the person’s notes that were reviewed to obtain this sort of information.
3. ***It is not possible to evaluate measures out of routine collection and documentation of timepoints of fetal pelvic entry, rumping, the emergence of the umbilical cord insertion, as well as timing of umbilical cord clamping and time to deliver are routinely not recorded in the birth report.*** – As we have answered above, we have reported transparently how often these were reported and could be included in our analysis, in each of our tables. Some practitioners at this site participated in training based on the Algorithm in 2018, and this may have influenced their awareness of the importance of recording these data points. Timing of umbilical cord clamping has been routinely recorded in the UK, since 2014 when NICE guidance recommended delayed cord clamping and listed it as an auditable criteria. Many other research studies have been done around timing of umbilical cord clamping. Recommendations on breech delivery timings are routinely made in obstetric textbooks. A key motivator for this study was that these recommendations are based on a lack of evidence at best, and some may be actively harmful. It is difficult to determine how to test these beliefs and develop a more robust evidence base without making some attempt to measure what these timings are in actual births.
4. ***Even recording the birth phase and pressing phase is subject to inaccuracies.*** – This is true. However, many obstetric midwifery guidelines do provide guidance on normal ranges for how long labour or pushing lasts and when it should be considered abnormal, when intervention is indicated. Almost all of the studies that underpin these recommendations are based on retrospective data collection, which is commonly understood to be less accurate than prospective data but the best we have to start with initially.
5. ***As long as these times are only indirectly estimated with unclear methodology, conclusions about the calculated periods are implausible*** **.** – We have reported transparently how often we were able to extract a time for each event from the maternity notes to include in our analysis. We used a structured data collection tool when reviewing the notes to extract this information. We have been clear in discussing the study limitations that these findings will need to be tested in further studies with a larger sample and prospective data collections, but we argue that the current study findings are of sufficient interest to disseminate on this basis.
6. ***Tools that were developed to estimate timepoints of delivery out of an imprecise or incomplete report have to be validated*** **.** –The tool is not designed to and was not used to estimate timepoints. It is a data collection tool. We extracted the data from the notes where they were reported. Our tool specified the data items that were extracted, which were also pre-specified in the protocol. We acknowledge that sometimes providers documenting care may have estimated timepoints when documenting in retrospect, but this would have been as accurate as any other maternity documentation.
7. ***Only a study with a prospective design and study protocol can accomplish this task*** **.** – This study provided important groundwork for a prospective study, trialling the use of the tool and establishing the value of a larger scale test of the hypotheses provided in the current study. As noted above, the study limitations were discussed and a prospective study is now in progress. However, there are inherent challenges in developing and conducting a prospective study of this topic – vaginal breech birth is now uncommon and the rate of adverse events implies an unfeasibly long time frame or involvement of a large number of services. In addition, the resources required to ensure fully accurate and complete prospective recording are considerable. These research challenges and the current state of lack of any formal evidence basis for current practice recommendations, in our view, demonstrate the value of sharing the findings of this study.

Some results are open or questionable:

1. ***Why are the pH-values in the tested group (=cases with 100% transfer rate ad NICU) higher than in the comparison group (=controls)? Early umbilical cord clamping as an argument is not plausible. Is the transfer to NICU due to reduced APGAR a result of manual intervention?*** – We also found this outcome surprising, leading us to have several conversations with experts who have conducted research on the timing of umbilical cord clamping and its relationship to cord gases. We intend to do further research in this area and have included it as an important data point in our prospective study, as it is also very important to service users. The questions arising from these results have also been discussed in the article.
  All neonatal admissions were following neonatal resuscitation, and this was documented as the reason for neonatal admission. This has now been clarified in the report of results. Our data indicated that manual interventions were initiated later on average among births where transfers to the NICU took place. In other reports of neonatal poor outcomes, such as the Term Breech Trial ( https://www.thelancet.com/journals/lancet/article/PIIS0140-6736(00)02840-3/fulltext ), complications of hypoxia outnumber complications of trauma. Therefore, we do not consider our data to indicate that earlier intervention, with appropriate technique, necessarily results in complications causing NICU admission. Concerns about trauma may be greater in other settings using different methods of delivery, and we intend to investigate this further in our on-going work.
2. **I *s a parameter to detect a higher neonatal transfer rate ad NICU with a sensitivity of 66% a good parameter to recommend consequences in delivery management? Is the collective of 15 cases perhaps too small?*** – Our recommendation pre-dates this study and came from a study of 42 videos ( https://onlinelibrary.wiley.com/doi/full/10.1111/birt.12480), in which the timings were recorded accurately, with precision. The purpose of this study was to test whether the recommendations in the Algorithm would help predict NICU admission. They did, but they more accurately predicted no NICU admission (the true negative rate).
  It is important that the reviewer’s dissent to this recommendation remain a part of the public record of this research. This dissent is consistent with the majority opinion in obstetrics, which has historically and in recent history suggested that much longer intervals are ‘safe’ in vaginal breech births and that intervention causes more injury than it prevents. It is also important that the basis for our recommendations are a matter of public record. We are testing them with a further multi-centre study and collecting the same data points in the OptiBreech Trial. If we are wrong, and prospective data collection is inconsistent with our findings or recommendations, this will soon become apparent.
3. ***What were the actual reasons for transferring the children to the NICU (immaturity? infection? hypoglycemia? Etc.)*** – We did not collect this information in this study and therefore cannot report it. All births occurred after 37 weeks; therefore immaturity was not a cause of admission. All neonatal admissions reported occurred immediately following birth; therefore hypoglycemia is unlikely to be a significant cause, as it is later in the neonatal period.
4. ***In how many births could the necessary data not be read out from the documentation, or could it be read incompletely or with uncertainty?*** – We have reported our denominators (the available data points) transparently in our tables. We only included parameters which were clearly documented or could be reliably inferred. For example, in a number of births the birth of the pelvis and the birth of the head occurred within the same minute, in which case the data collector considered it reliable to assume that the birth of the umbilicus occurred within the same minute.
5. ***Rapid, simple vaginal birth always tends to have a better outcome for the children, while protracted birth leads to a poorer outcome, with and without intervention. What weighs more heavily for the newborn poor outcome from intervention and/or acidosis?*** – We agree with the first statement, although our experience and the evidence in this study indicates that intervention can often be rapid, simple and effective at preventing protracted births. In our OptiBreech trial protocol, encouraging maternal movement and effort is the first recommended intervention; in current practice, this is often avoided, and women are instructed instead to, “Breathe and wait for the next contraction.” In the Term Breech Trial, complications of asphyxia were more common than traumatic neonatal injuries. In the PREMODA study ( https://www.ajog.org/article/S0002-9378(05)02440-3/fulltext), systematic manoeuvres were used routinely in 30.3% of births, with much better outcomes than those reported in the Term Breech Trial. While we are not arguing that every birth requires systematic manoeuvres, we feel that assistance is wrongly avoided in many breech births and that harm results from this avoidance. Our trial protocol also recommends a lower threshold for intervention than current national guidelines in the UK. The evidence in this report partly explains why.
6. ***As long as the collection of the data at the calculated times (timeline) is not comprehensible, this study cannot be approved for indexing in this form*** *.* – We respectfully ask the reviewer to clarify what would make collection of this data comprehensible. We readily accept that use of a pro forma for documentation purposes, as we are doing in the OptiBreech Trial, has made it somewhat easier. However, documentation of key points, such as when buttocks are visible or born, when legs and arms are born, when the head is born and when the umbilicus is cut, is considered best practice already in UK maternity care.

The reviewer has answered ‘no’ to the question, "Are sufficient details of methods and analysis provided to allow replication by others?". We understand the concerns were related to how data points were extracted from the notes, and we have provided clarification around this in our description of methods. We are replicating the study at multiple sites following a similar protocol and welcome others to do the same. Others may find different results or interpret them differently. We encourage replication and publication of this topic. If we are incorrect, and earlier intervention causes harm, it is important that this be apparent as soon as possible. If we are correct, and babies would benefit from earlier intervention to prevent unnecessary hypoxia, further evidence will clearly be needed to achieve a change in practice.
